# Predictions of time to HIV viral rebound following ART suspension that incorporate personal biomarkers

**DOI:** 10.1371/journal.pcbi.1007229

**Published:** 2019-07-24

**Authors:** Jessica M. Conway, Alan S. Perelson, Jonathan Z. Li

**Affiliations:** 1 Department of Mathematics and Center for Infectious Disease Dynamics, Pennsylvania State University, University Park, Pennsylvania, United States of America; 2 Theoretical Biology and Biophysics, Los Alamos National Laboratory, Los Alamos, New Mexico, United States of America; 3 Brigham and Women’s Hospital, Harvard Medical School, Boston, Massachusetts, United States of America; Emory University, UNITED STATES

## Abstract

Antiretroviral therapy (ART) effectively controls HIV infection, suppressing HIV viral loads. Suspension of therapy is followed by rebound of viral loads to high, pre-therapy levels. However, there is significant heterogeneity in speed of rebound, with some rebounds occurring within days, weeks, or sometimes years. We present a stochastic mathematical model to gain insight into these post-treatment dynamics, specifically characterizing the dynamics of short term viral rebounds (≤ 60 days). Li et al. (2016) report that the size of the expressed HIV reservoir, i.e., cell-associated HIV RNA levels, and drug regimen correlate with the time between ART suspension and viral rebound to detectable levels. We incorporate this information and viral rebound times to parametrize our model. We then investigate insights offered by our model into the underlying dynamics of the latent reservoir. In particular, we refine previous estimates of viral recrudescence after ART interruption by accounting for heterogeneity in infection rebound dynamics, and determine a recrudescence rate of once every 2-4 days. Our parametrized model can be used to aid in design of clinical trials to study viral dynamics following analytic treatment interruption. We show how to derive informative personalized testing frequencies from our model and offer a proof-of-concept example. Our results represent first steps towards a model that can make predictions on a person living with HIV (PLWH)’s rebound time distribution based on biomarkers, and help identify PLWH with long viral rebound delays.

## Author summary

Antiretroviral therapy (ART) effectively controls HIV infection, holding HIV viral loads to levels undetectable by commercial assays. Therapy interruption is followed by rebound of viral loads to high, pre-therapy levels, but there is significant heterogeneity in the timing of the rebound to those high levels. Some rebounds occur within days, weeks, or even, rarely, years. Here we develop a mathematical model to characterize rebounds occurring within two months of treatment interruption. Li et al. (2016) report biological markers that correlate with the time between ART interruption and viral rebound. We incorporate this information to parametrize our model so that our model can make predictions on time to rebound tailored to the individual undergoing ATI. Our parametrized model can aid in design of clinical trials to study infection dynamics following treatment interruption. We also use our model to gain insight into the underlying within-host viral dynamics. For example, we refine previous estimates of viral recrudescence after ART interruption and determine a recrudescence rate of once every 2-4 days. Our results represent first steps towards a model that can make predictions on an person living with HIV’s rebound time based on personal biomarkers, and help identify patients with long viral rebound delays.

## Introduction

Antiretroviral therapy (ART) for HIV infection can very effectively control the infection and hold the amount of circulating virus below the level detectable by clinical assays, improving both the quality and length of life. ART suspension generally is followed by HIV rebound to high viral loads [1], and consequently the standard of care for people living with HIV (PLWH) is to maintain life-long ART. However, there is significant heterogeneity in rebound times. In a pooled analysis of participants from six AIDS Clinical Trials Group (ACTG) analytic treatment interruption (ATI) studies to identify predictors of viral rebound, Li et al. reported widely varying times to viral rebound, with a significant number of participants maintaining viral suppression to undetectable levels for up to 2 or more months in the absence of ART [2]. In a follow-up study, Li and his team identified a cohort of post-treatment controllers (PTCs) from these ATI studies, who maintained viral loads ≤ 400 HIV RNA copies/mL for ≥24 weeks [3, 4]. Previous reports of these rare PTCs include the VISCONTI cohort, 14 PLWH who initiated ART within three months of their estimated date of infection who were able to control HIV infection for a prolonged period after stopping ART [5]. Results from the VISCONTI study and others suggest that PTCs may control HIV by a mechanism distinct from that of spontaneous HIV controllers [6, 7]. However, the factors that mediate delayed timing of HIV rebound are not well understood.

Since ART comes with a number of drawbacks including side-effects and cost, the search for biological indicators (biomarkers) of lasting ART-free HIV remission has become a priority in HIV cure research [8, 9]. Studies have already begun to bear fruit, with recent studies revealing a variety of immunological biomarkers for delayed rebound and infection control [2, 3, 10–13]. While such studies are informative, they offer limited insight into the mechanisms underlying viral rebound or post-treatment control. Mechanistic modeling inference has an established history of advancing our understanding of HIV [14, 15]. In this study we combine data on markers associated with rebound identified by Li et al. [2] with mechanistic mathematical models to gain deeper insight into mechanisms of viral rebound.

Modeling within-host HIV infection and treatment is a well-established field [14, 16–24]. By fitting models to clinical data, many parameters describing HIV dynamics such as the viral clearance rate, the infected cell death rate, and the viral burst size have been estimated [25–27]. Existing models have mainly focused on the kinetics of early infection and the effects of treatment. A few papers have focused on HIV control and the time to viral rebound after treatment cessation. These include those of Hill et al. [28, 29], Pinkevych et al. [30, 31] and Fennessey et al. [32], in which the authors all assume viral rebound is the outcome of latent cell activation. Pinkevych et al. used data from treatment interruption trials to provide the first estimates of latent cell activation rates that lead to observable viremia [30, 31]; using a related approach Fennessey et al. investigated SIV viral rebound in macaques infected with barcoded virus, to generate more detailed insights into viral rebound [32]. However, this modeling does not account for individual-level heterogeneity in viral rebound dynamics [33]. Hill et al. used continuous time branching processes, which are well-suited for small populations, to model within-host viral rebound dynamics. The primary results in Hill et al. [28, 29] are estimates of viral rebound time distributions, used in combination with careful and thoughtful consideration of within-host parameters to evaluate the needed efficacy of therapeutic agents that one day may be able to reduce the latent reservoir. In their model, Hill et al. assumed that latently infected cells may die or activate and that newly activated cells can die or generate infected cell offspring that are infected, as a proxy for tracking virus that in turn infects new cells [28, 29]. Thus, Hill et al. assumed that average viral growth immediately following viral recrudescence is, on average, exponential, which may not be the case.

In this study, we take up the hypothesis that latent cell activation causes viral rebound in the short term (<60 days) [28, 29], but that significantly delayed rebounds are associated with additional mechanisms of infection control, such as anti-HIV immune responses [23]. For example, there is evidence that T-cell exhaustion markers are predictive of shorter time to viral rebound [13] and that levels of HIV-specific T cell responses is associated with viral load after ATI [11]. We focus on short-term delays. We fit a simple stochastic model of viral rebound extended from our previous studies [21, 24] to the viral rebound data from the ACTG ATI studies [2]. In contrast to Pinkevych et al. [30] we address uncertainty in latent reservoir rebound dynamics, by modeling the time between a “successful” latent cell activation and detectable viremia stochastically and given by one of a variety of probability density functions. We integrate into our model biomarkers with observed impact on time to viral rebound, e.g., an individual’s expressed HIV reservoir, i.e., levels of cell-associated HIV RNA (HIV CA-RNA or CA-RNA), and ART regimen pre-analytic treatment interruption (ATI) [2, 34]. We discuss biological insight offered by parameter estimates from data, in particular on the average rate of latent cell activations that cause viral rebound [30, 31, 33]. Our model output is a cumulative probability density function for the probability of an individual’s viral rebound at time *t*. The output can be used for ATI clinical trial design; in particular, one can derive from our modeling a viral load testing schedule for participants to meet study objectives.

## Materials and methods

Our aim is to construct a model that predicts the viral rebound time, i.e., the time between suspension of therapy and detectable viremia. We model viral dynamics following cessation of therapy using the central assumption that activation of latently infected cells drives viral rebound. To estimate model parameters, we use data collected from ACTG ATI studies.

### Ethics statement

Written informed consent was provided by all study participants for use of stored samples in HIV-related research. This study was approved by the Pennsylvania State University Institutional Review Board, the Los Alamos National Laboratory Institutional Review Board, and the Partners Institutional Review Board.

### Description of data

The description of the data we employ and associated collection methodologies are fully explained in [2]. Briefly, participants in six ACTG ATI studies (ACTG 371 [35], A5024 [36], A5068 [37], A5170 [38], A5187 [39], and A5197 [40]) were included if they were on suppressive ART, received no immunologic interventions (e.g., therapeutic vaccination, interleukin-2), and had HIV-1 RNA less than 50 copies/ml at the time of ATI (*N* = 235 participants). We restricted the data we analyzed to the participants who showed viral rebound ≤ 60 days after ART cessation, in part to accommodate model simplifications, such as our assumption that the latent reservoir size remained constant from ATI to the time of viral rebound (see **Model**, below). Of the *N* = 210 participants who rebounded within 60 days of ATI, *N* = 84 met the following additional criteria for further study: 1) had peripheral blood mononuclear cells (PBMCs) and plasma available for HIV reservoir quantification while on ART prior to the ATI and 2) had cell-associated HIV-1 RNA (HIV CA-RNA) above the level of detection at ATI. Cell-associated DNA was also measured but did not have a significant association with time to viral rebound [2] and is neglected in our analysis. Finally, Li et al. noted that viral rebound delays were greater in study participants whose pre-ATI ART regimen contained non-nucleoside reverse transcriptase inhibitors (NNRTIs) [2]. We therefore distinguish between regimens containing a NNRTI (50/84 study participants) and those that do not (34/84 study participants).

Early after treatment interruption, most studies reported weekly viral load measurements, with the exception of A5170. In total, in the subset of study participants we study here (*N* = 84/235), 41 participants had approximately weekly or more frequent viral load measurements, while the remaining participants had viral loads measured more frequently than monthly, with a median of 7 days (range 1-35 days; interquartile range (IQR) 6-24 days). The timing of viral rebound was defined as sustained viral loads of at least 200 HIV RNA copies/mL.

**Fig 1:**
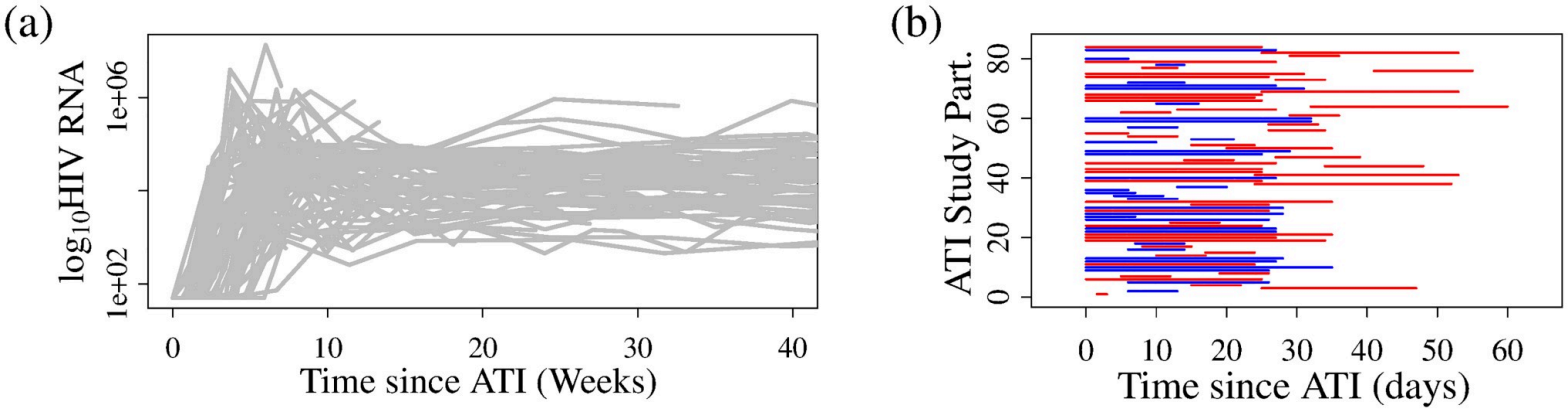
Participant viral loads following ATI. (a) Plasma HIV RNA levels following ATI; each corner represents a measurement, with lines used to connect the measurements from the same participant. (b) The times of last undetectable measurement and first detectable measurement shown as line segments spanning the detection window per ATI study participant, with color indicating whether the pre-ATI ART regimen included (red) or excluded (blue) NNRTIs.

Viral load data is shown in Fig 1a. In this present study we model the time of viral rebound, which occurs at some point between a study participant’s last undetectable and first detectable viral load measurement (threshold of detection 200 HIV RNA copies/mL). We will therefore use those time points to estimate model parameters for each ATI study participant. The times of last undetectable measurement and first detectable measurement are shown in Fig 1b as line segments spanning the detection window per study participant, with color indicating whether the ART regimen included (red) or excluded (blue) NNRTIs. Although the median time between viral load tests up to the time of detectable viremia, across the 84 study participants, is 7 days, the median time window between the last undetectable measurement and the first detectable viral load measurement, shown in Fig 1b, is 20 days (range 4-35 days; IQR 7-27 days).

### Model

**Fig 2:**
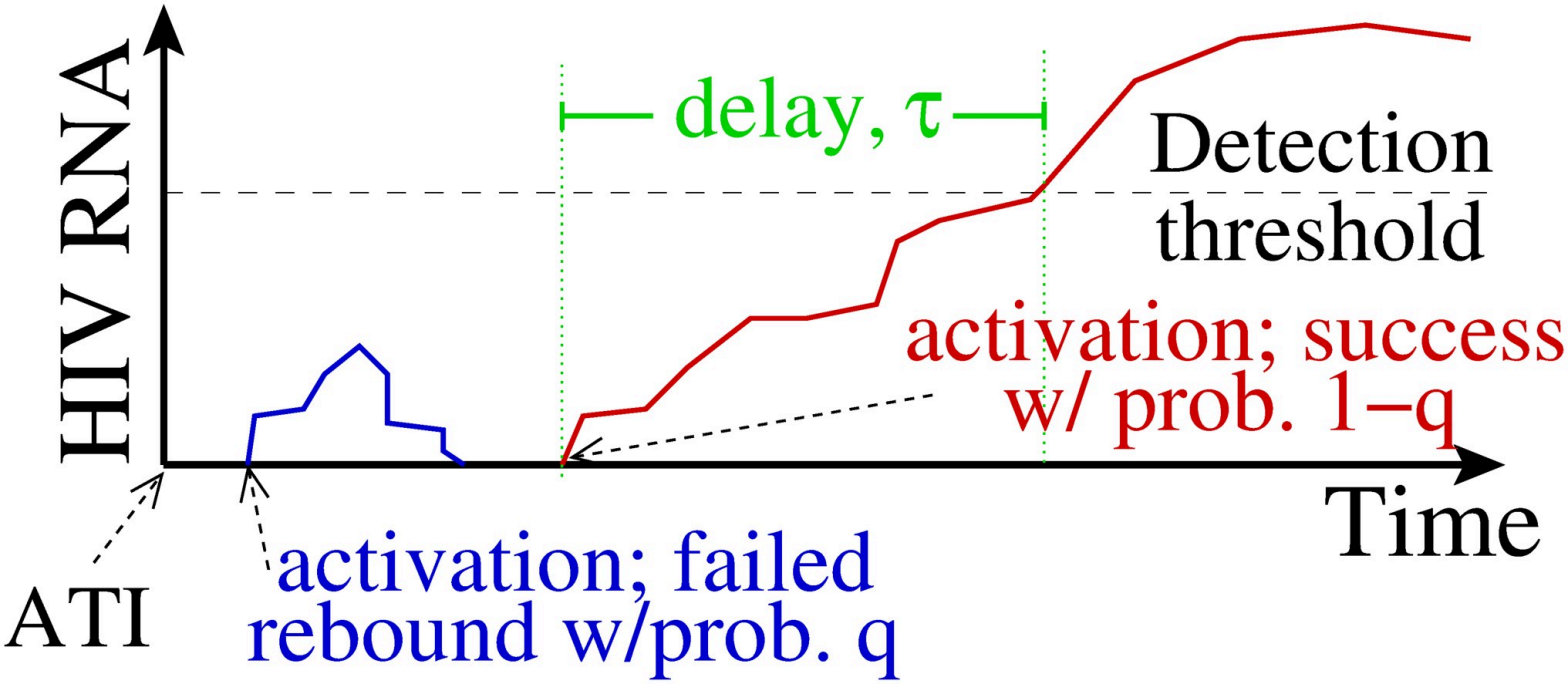
Model schematic. We assume that following ATI, latent cell activations are followed by chains of infection that may die out, i.e., go extinct, with probability *q*, or successfully re-establish high viral loads associated with chronic infection, with probability 1 − *q*. In the latter case, we further assume a delay *τ* between activation and the time when plasma viral load crosses the detection threshold.

We assume that activation of latently infected cells drives viral rebound [29, 30] and model the ensuing dynamics as illustrated in Fig 2. Not all latently infected cell activations cause viral rebound. We assume that activation is followed by rounds of viral replication, which may cause viral populations to grow to detectable levels, thereby causing viral rebound, but may also die out. We define *q* as the probability of extinction, i.e., the probability that the rounds of viral replication following latent cell activation die out, that is, that the activation of a latently infected cell does not cause viral rebound. We further define a “successful latent cell activation” as one that does cause viral rebound. In the time preceding a successful latent cell activation, we envision viral dynamics similar to those modeled in [24], with potentially many latent cell activations followed by a few rounds of viral replication, with the lineages ultimately going extinct. We also assume that any activations pre-ATI and resultant lineages go extinct as drug is still restricting viral spread (see **Incorporating rebound indicators** and and Discussion).

Finally, we assume that there is a delay between successful latent cell activation and detectable infection, where “detectable infection” corresponds to study participant viral loads exceeding 200 HIV RNA copies/mL. There is some debate as to the dynamics following latent cell activation. For example, *in vitro* observations suggests that an activated latently infected cell may produce significantly less virus than a productively infected cell [41]. Further, following the application of latency reversing agents, latently infected cells dynamics may not conform to the observed dynamics of productively infected cells [42], and latently infected cells may divide before they get fully activated and produce virus [28, 41]. We therefore avoid the common assumption that an activated latently infected cell is the same as a productively infected cell [21, 24, 28]. The need of target cells to infect in the proximity of an activated latently infected cell may also pose a challenge in initiating detectable infection *in vivo*. Finally, heterogeneity in viral growth rates (once there is enough virus for exponential growth) among individuals, due to difference in the infecting virus and host restriction factors, is also a factor in the early dynamics [30, 31, 33]. Because the dynamics of latent cell activation and infection spread before a detectable level of viremia is attained are unknown, we absorb these dynamics into a delay-time distribution, *D*(*t*) (Fig 2) reflecting these various sources of heterogeneity. We will test differing assumptions on this delay, for example taking a fixed or distributed delay (e.g. a lognormal distribution).

We restrict ourselves for now to short-term viral rebound (≤ 60 days). Over that short time period, we can assume that the latent reservoir size is approximately constant with value *L*_0_ (<3% estimated reduction, assuming that while the virus is undetectable the latent reservoir continues to decay with a half-life of 44 months [43, 44]). We assume that latently infected cells are activated at an average rate *a*, so activated cell influx occurs at the constant rate *aL*_0_. In general, we anticipate variability in the activation rate *a*; for example, most latently infected cells are memory cells [45], and activation may depend on encounters with cognate antigen, whose rates may be expected to vary according to the rarity of the associated pathogen. However, per the law of large numbers, given the large latent reservoir size in most individuals [46], the time to detection will approximately depend on the average activation rate. Mathematically we employ a multi-type branching process framework to derive an expression or viral rebound at time *t*. We use this probability to ultimately derive likelihood functions and fit the model to data.

To sum up, in our model we assume that heterogeneity in observations of viral rebound across individuals result from four components. Two depend on the individual study participant and derive from observed correlates of time to viral rebound: (i) The replication-competent reservoir size *L*_0_, which we will assume is reflected in the HIV CA-RNA level [2, 34], and (ii) the probability *q* that the activation of a latently infected cell does not cause viral rebound, which may be affected by the pre-ATI ART regimen [2]. The remaining two components arise from stochastic within-host dynamics: (iii) the rate of latent cell activations that are “successful”, which we model as a Poisson rate, and will result in an exponential distribution in time to first successful activation, and (iv) the stochastic delay between successful activation and detection, which we model using different stylized distributions.

#### Cumulative probability of viral rebound at time *t*, *P*_*VR*_(*t*)

From a simple branching (immigration) process, given *aL*_0_(1 − *q*), the influx of activations that induce viral rebound, we can compute the cumulative probability of a successful activation at time *t* as *P*_*ACT*_(*t*) = 1 − *Prob*(*A*(*t*) = 0), i.e., subtracting from 1 the probability that at time *t* there have been no successful activations, *A*(*t*). We recover *P*_*ACT*_(*t*) = 1 − exp(− (1 − *q*)*aL*_0_*t*) (see S1 Text for details on the derivation). Then, assuming that there is a delay between a successful activation and development of detectable viremia, *D*(*t*), the probability of viral rebound at time *t* predicted by our model is

$$\begin{array}{c} P_{VR}\left(t\right)=\int_{0}^{t}(1-e^{-\left(1-q\right)aL_{0}\left(t-\tau \right)})D\left(\tau \right)\;d\tau \end{array}$$

(see S1 Text). We will test different forms of the delay distribution *D*(*t*).

#### Delay distribution *D*(*t*)

The delay distribution, *D*(*t*), is intended to describe the unknown dynamics between a successful latent cell activation and viral detection and is assumed to be shared across individuals. We will test several stylized distributions over the positive real axis to model the delay: fixed (delta-distributed), exponentially-distributed, gamma-distributed, lognormally-distributed, Weibull-distributed, and log-logistically distributed delays. The probability density functions and associated parameters to be estimated are given in Table 1. We include the exponential distribution for completeness. However we note that, for our purposes, it is biologically infeasible as it would permit, with highest probability, no delay between successful activation and infection detection.

**Table 1:** Stylized delay distributions, *D*(*t*).

Delay distribution	Probability density function *D*(*t*)	Parameters
Fixed (Delta-distributed)	*δ*(*t* − *t*_delay_)	fixed delay time *t*_delay_
Exponential	λ*e*^−λ*t*^	rate λ
Gamma	*β*^*α*^*t*^*α* − 1^ *e*^−*βt*^/Γ(*α*)	shape *α*, rate *β*
Lognormal	$\exp[-{\left(\ln t-\mu \right)}^{2}/2\sigma^{2}]/t\sigma \sqrt{2\pi }$	mean *μ* and st. dev. *σ* of logarithm of *t*
Weibull	$(\kappa /\lambda ){\left(t/\lambda \right)}^{\kappa -1}e^{-{\left(t/\lambda \right)}^{\kappa }}$	scale λ, shape *κ*
Log-logistic	(*β*/*α*)(*t*/*α*)^*β*−1^/(1 + (*t*/*α*)^*β*^)^2^	scale *α*, shape *β*

#### Incorporating rebound indicators

Li et al. [2] analyzed data from 235 ATI study participants (see **Description of data**) to determine predictors of viral rebound timing. Their univariate analysis showed that factors significantly associated with earlier timing of viral rebound included levels of cell-associated RNA (CA-RNA), presence of detectable residual HIV viremia using a single-copy assay, and the use of non-nucleoside reverse transcriptase inhibitor-containing drug regimens. The effect of confounding was evaluated by a 2-covariate Cox model of CA-RNA with other variables as predictors of viral rebound timing including ART regimen and detectable residual viremia. The odds ratio for CA-RNA remained stable regardless of the model, and both detectable viremia and ART regimen remained significant when combined in models with CA-RNA levels [2]. Intriguingly, no significant association was found between levels of cell-associated HIV DNA (CA-DNA) and the timing of viral rebound [2], perhaps due to the large fraction of latently infected cells that have defective proviruses [46]. We therefore do not use CA-DNA as a measure of the replication competent latent reservoir.

**Fig 3:**
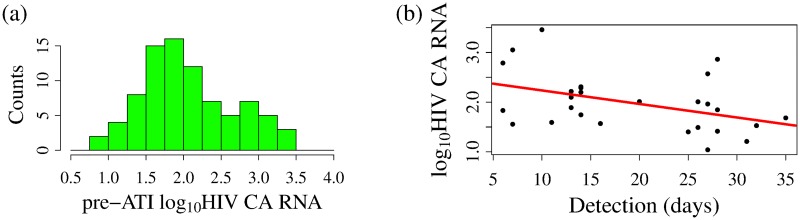
HIV CA-RNA and HIV viral rebound. (a) Histogram showing pre-ATI log_10_(HIV CA RNA (per 10^6^ CD4+ cells)) across study participants. (b) Correlation between pre-ATI log_10_(HIV CA-RNA (per 10^6^ CD4+ cells)) and time to viral rebound in non-NNRTI study participants (p-value 0.0260). Data shown only for study participants who showed viral loads less than 10000 HIV RNA/mL at first detection.

We incorporate pre-ATI CA-RNA levels into our model by assuming that the reservoir size per study participant is proportional to the log of the CA-RNA level, i.e., *L*_0_ = *s* log_10_(CA-RNA). Li et al. demonstrated a significant association between pre-ATI HIV CA-RNA and a shorter time to viral rebound using a different approach, grouping study participants by time to viral rebound, and using the Kruskal-Wallis test [2]. We justify the use of a correlation with the logarithm instead with the observation of an order of magnitude variability in RNA per cell [47] and the reasonable correlation that we find between log_10_(HIV CA-RNA) and the time to detectable viremia. The distribution of the log_10_(HIV CA-RNA) across study participants is shown in Fig 3a; the time to first detectable viral load measurement per study participant is negatively correlated with log_10_(CA-RNA), see Fig 3b. Note that the first detectable viral load measurement is not equivalent to the time the viral load first crosses the detection threshold; however, since the measurement follows rebound we find the correlation suggestive. Using the formula *L*_0_ = *s* log_10_(CA-RNA), the probability of viral rebound in an ATI study participant, *P*_*VR*_(*t*), becomes

$$\begin{array}{c} P_{VR}\left(t\right)=\int_{0}^{t}(1-e^{-\left(1-q\right)as\;{\text{log}}_{10}(\text{CA-RNA})\left(t-\tau \right)})D\left(\tau \right)\;d\tau \end{array}$$ (1)

**Fig 4:**
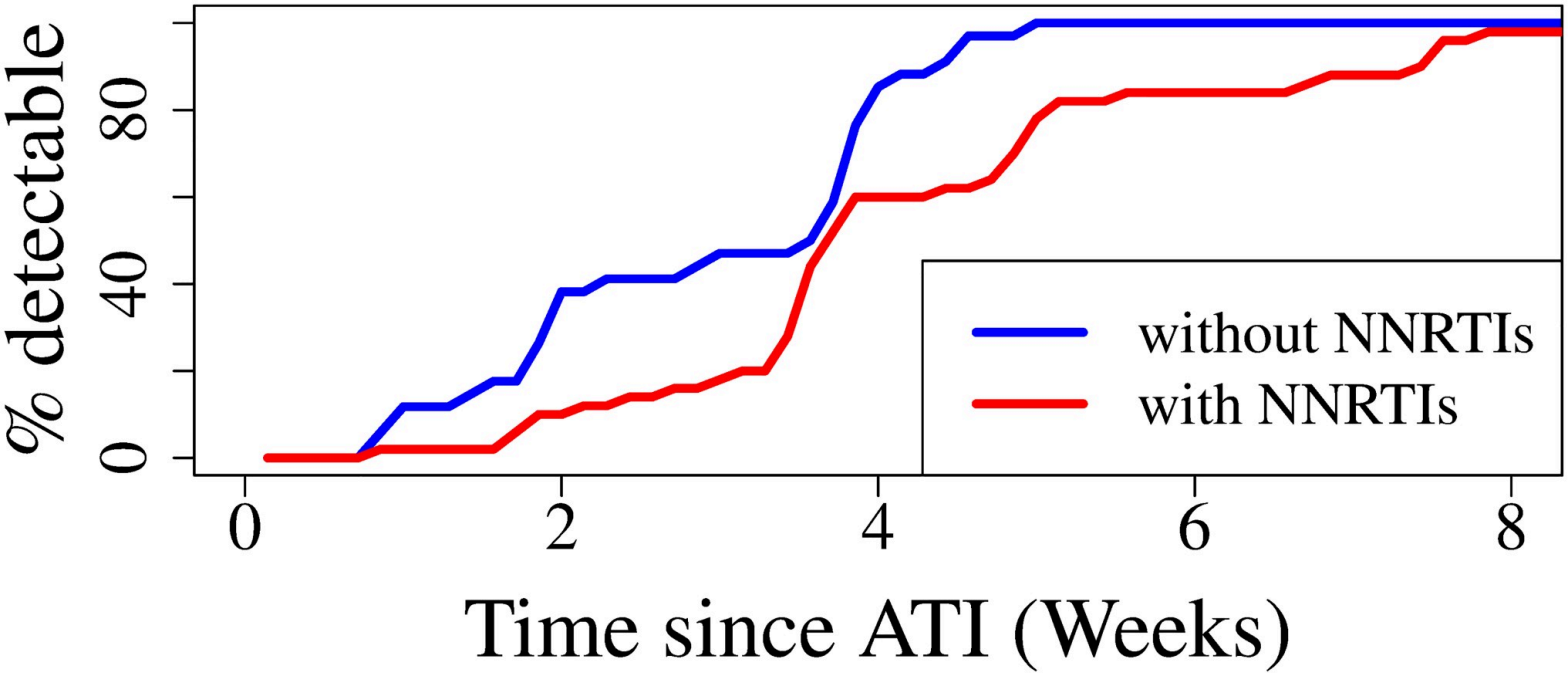
Time post-ATI of first detectable viral load measurement depending on study participant drug regimen, with or without NNRTIs. Note the statistically significant delay in viral rebound in participants taking NNRTIs pre-ATI (Wilcoxon rank sum test; p-value<0.01).

Fig 4 shows the empirical distribution of the time to first detectable viral load measurement for study participants, separated according to pre-ATI ART regimen from [2], demonstrating the statistically significant delay in viral rebound in participants taking NNRTIs pre-ATI (Wilcoxon rank sum test; p-value<0.01). As discussed in Li et al. [2], the delay is likely due to the significantly longer half-life of the NNRTI class of medications. We model the delaying effect of NNRTI washout through the probability that an activation does not induce rebound, *q*, since drugs inhibit viral replication and should thus enhance this probability. We assume that *q* ≡ *q*(*t*) = *q*_0_ + (1 − *q*_0_)*e*^−*kt*^. The exponential decay assumption is consistent with decay of efavirenz after treatment interruption [48], and with standard pharmacokinetic/pharmacodynamic modeling [49, 50]. Our crude approximation can be derived from pharmacodynamic models, e.g. [50], with some simplification (see S1 Text). In our formulation for *q*(*t*), the probability of extinction at the at the time of ATI (*t* = 0) is 1, since no activations induce rebound in the presence of effective therapy. Following interruption of therapy (*t* > 0), the probability of not inducing rebound exponentially decays at rate *k* to *q*_0_, 0 < *q*_0_ < 1. This decaying effect should be present with any ART drug, and not just NNRTIs. However, the decay is much more rapid with other drug types [51] and our data during the first few days following ATI is sparse, making the rapid decay very difficult, if not impossible, to detect. Therefore, for simplicity the rapid decay of non-NNRTI drug therapy is ignored here. With this time-dependent probability that an activation will cause rebound, *q*(*t*), we can re-derive the probability of viral rebound, yielding

$$\begin{array}{c} P_{VR}^{\text{NNRTI}}\left(t\right)=\int_{0}^{t}\{1-\exp[-\frac{as\log_{10}(\text{CA-RNA})\left(1-q_{0}\right)(\text{exp}\left(-k\left(t-\tau \right)\right)-1+k\left(t-\tau \right)}{k}]\}D\left(\tau \right)\;d\tau \end{array}$$

(see S1 Text). Thus, if we want to account for the presence or absence of NNRTIs in the ART regimen,

$$\begin{array}{c} \;P_{VR}^{}\left(t\right)=\{\begin{array}{cc} \int_{0}^{t}(1-e^{-\left(1-q\right)as\log_{10}(\text{CA-RNA})\left(t-\tau \right)})D\left(\tau \right)\;d\tau & \text{in}\;\text{the}\;\text{absence}\;\text{of}\;\text{NNRTIs}\\ \int_{0}^{t}\{1-\exp[-\frac{as\log_{10}(\text{CA-RNA})\left(1-q_{0}\right)(\text{exp}\left(-k\left(t-\tau \right)\right)-1+k\left(t-\tau \right)}{k}]\}D\left(\tau \right)\;d\tau , & \text{in}\;\text{the}\;\text{presence}\;\text{of}\;\text{NNRTIs} \end{array} \end{array}$$ (2)

We will test models accounting for, and neglecting, NNRTI use in ATI study participants and compare model fits using the Akaike information criterion [52].

Finally, we neglect the role of detectable residual viremia in study participants on ART. Implicitly we are neglecting any activations before ATI and resultant rounds of viral replication. We argue that this neglect isn’t fatal. In the presence of effective and even waning ART, modeling suggests only very few rounds of replication are likely [24], yielding extinction for lineages initiated by an activation pre-ATI on the same or shorter time scale as ART decay. Thus we assume that lineages generated by activations pre-ATI go extinct. However, we note that this last assumption is empirically untested. Li et al. (2016) observed a correlation between time to viral rebound and pre-ATI viral load [2], and Kearney et al. noted that rebound HIV in plasma may be sourced from cells that were transcriptionally active, i.e., replicating, before ATI [53]. These observations are not inconsistent with our modeling assumptions, since the source of rebound post-ATI may be activations from the same clone as residual viremia shortly before ATI, and resultant pre-ATI viral loads would still depend on *aL*_0_. But we cannot discount alternative hypotheses.

#### Likelihood function, $\text{lik}(\vec{\theta })$

We can use the model-derived probability of viral rebound at time *t*, either Eqs (1) or (2), to derive a likelihood function that we can maximize to estimate parameters. Our data, described in **Description of data**, gives only the time post-ATI of the tests with the last undetectable, and first detectable, viral load for each study participant. Our model predicts that the probability of rebounding in that time window is *P*_*VR*_(First detectable test) − *P*_*VR*_(Last undetectable test). We define this difference as our likelihood, given the parameter set $\vec{\theta }$,

$$\begin{array}{c} \text{Likelihood}\left(\vec{\theta }\right)=\prod_{j=1}^{84}(P_{VR}\left(\text{First}\;\text{detectable}\;\text{test}\;\text{for}\;j\right)-P_{VR}\left(\text{Last}\;\text{undetectable}\;\text{test}\;\text{for}\;j\right)), \end{array}$$

multiplied over all study participants *j*. To estimate parameters, we maximize the likelihood with respect to model parameters, using the Davidon-Fletcher-Powell optimization algorithm in R [54].

We estimate *k*, *as*(1 − *q*_0_), and any parameters associated with the delay distribution *D*(*t*) (cf. Table 1). Note that the parameters *a*, *s*, and *q*_0_ cannot be separately estimated as they appear in Eqs (1) and (2) only as the parameter combination *as*(1 − *q*_0_), i.e., the scaled influx of activations that induce viral rebound. This should not be a surprise, because the data and the theory deal with viral rebounds which result from successful activations only.

## Results

We begin by estimating model parameters. We then go on to discuss the consequences of our estimates, in particular the impact of NNRTI-containing drug regimens on viral rebound times, and our estimates of successful latently infected cell activation times. Finally, we discuss how our model predictions can be used to inform clinical trial design.

### Model parameter estimation; accounting for NNRTI use significantly improves quality of fits

We use the Davidon-Fletcher-Powell optimization algorithm to estimate model parameters *as*(1 − *q*_0_), *k*, and parameters associated with the delay distribution, for our viral rebound model that neglects NNRTI status, Eq (1), and accounts for NNRTI status, Eq (2). A summary of these parameter estimates are provided in Tables 2 and 3, respectively, with complete details provided in S1 and S2 Tables, respectively. We use the Akaike Information Criterion (AIC) to compare how well the models explain the data.

**Table 2:** Key parameter estimates for model (1), making no distinction between participants based on pre-ATI ART regimen, with the 95% confidence interval indicated in parentheses. All parameter provided in S1 Table.

Delay type	*as*(1 − *q*_0_) (per log_10_ CA-RNA per day)	Mean delay (days)	AIC	Neg. log-likelihood
Fixed	0.04 (0.03, 0.06)	4.6 (3.4, 6.3)	180.3	88.1
Exponential	0.08 (0.04, 0.15)	9.5 (5.6, 14.5)	176.4	86.2
Gamma	0.29 (0.004, 19.831)	14.7 (8.5, 25.4)	175.9	85.0
Lognormal	0.08 (0.03, 0.2)	10.5 (5.0, 17.8)	178.2	86.1
Weibull	0.25 (0.01, 2.78)	14.5 (8.2, 20.8)	175.4	84.7
Log-logistic	0.07 (0.03, 0.14)	9.2 (2.0, 21.6)	179.8	86.9

**Table 3:** Key parameter estimates for model (1) with the 95% confidence interval indicated in parentheses, distinguishing study participants based on pre-ATI ART regimen, specifically inclusion of NNRTIs. Distribution-specific parameter estimates provided in S2 Table.

Delay type	*as*(1 − *q*_0_) (per log_10_ CA-RNA per day)	Decay rate *k* (per day)	Mean delay (days)	AIC	Neg. log-lik.
Fixed	0.10 (0.06, 0.17)	0.03 (0.02, 0.07)	4.2 (2.7, 6.2)	156.1	75.1
Exponential	0.14 (0.06,0.30)	0.03 (0.01,0.07)	5.2 (2.6, 9.3)	155.7	74.9
Gamma	0.22 (0.04,0.67)	0.02 (0.004,0.076)	6.8 (3.8, 11.9)	155.1	73.6
Lognormal	0.18 (0.04,0.53)	0.02 (0.01,0.07)	6.4 (2.6, 9.0	155.7	73.9
Weibull	0.24 (0.04,1.21)	0.02 (0.003,0.07)	7.0 (4.0, 12.0)	154.8	73.4
Log-logistic	0.16 (0.05, 0.50)	0.02 (0.01,0.07)	6.1 (3.1, 11.5)	156.3	74.2

#### We cannot determine the most appropriate detection-delay time distribution

Before discussing model selection, we observe, for the models that include or neglect NNRTI status, that fits under different delay distribution assumptions, *D*(*t*), are statistically indistinguishable as judged by the AIC (Tables 2 and 3). Our inability to discern a best-fit delay distribution is because the data is not sufficiently refined; mean delay estimates, depending on the model, range from roughly 4 to 23 days. But the median detection window—time between the last undetectable, and first detectable, viral load tests following ATI—is 20 days, with a mean of approximately 18 days; therefore the observations are not sufficiently frequent to characterize a distribution about the mean.

#### Model selection

**Fig 5:**
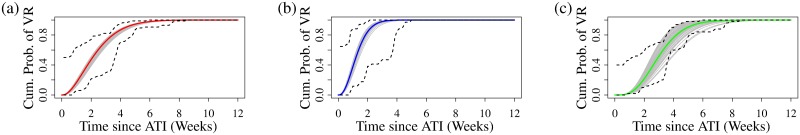
Model predictions on time to viral rebound (VR). Model predictions on time to VR for each ATI study participant (thin grey line) and the average time to viral rebound (thick, solid line), for (a) parameters estimated across all participants while neglecting pre-ATI ART regimen, and participants whose pre-ATI regimen (b) excluded or (c) included NNRTIs. The black, dashed curves give the empirical distributions of the time of last undetectable viral load test and first detectable viral load test.

Fig 5 gives the model-predicted cumulative probability of viral rebound at time *t*. Fig 5a shows predictions neglecting the effect of the pre-ATI ART regimen, using Eq (1), while Fig 5b and 5c shows the predictions accounting for the pre-ATI ART regimen, using Eq (2), for study participants whose regimen excluded NNRTIs (Fig 5b) or included NNRTIs (Fig 5c). Since we incorporate the individual’s pre-ATI HIV CA-RNA levels and NNRTI status, the models make predictions for each individual, shown as thin grey lines in all panels of Fig 5. Fig 5 also gives, in heavy lines, the population mean associated with each model, and, in dashed lines, the empirical cumulative distribution functions for the times of the last undetectable viral load test and the first detectable viral load test. Individual predictions when accounting for NNRTI status, Fig 5b and 5c, better the data than individual predictions neglecting NNRTI status, Fig 5a. We make this conclusion based on the model AICs: in computing AICs for our model fits neglecting (Table 2) or accounting (Table 3) for NNRTI status we see immediately that there is strong support for the latter models, which have AIC values that are at least 10 points lower than the values in Table 2 [52].

**Fig 6:**
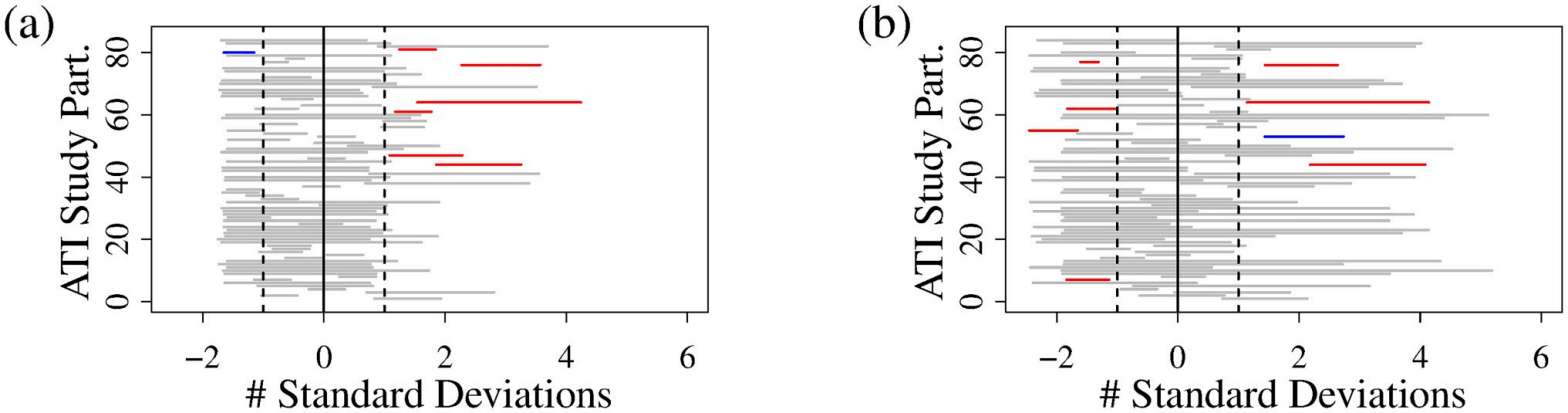
Visualization of model predictions. Visualization of model predictions (a) neglecting pre-ATI ART regimen, using Eq (1), and (b) accounting for pre-ATI ART regimen, using Eq (2). The normalized times of last undetectable measurement and first detectable measurement are shown as line segments spanning the detection window per ATI study participant. Each detection window’s line segment is normalized by subtracting the associated model-predicted mean and dividing by the model-predicted standard deviation. Red and blue lines indicate detection windows that lie wholly outside one standard deviation, with color indicating whether the pre-ATI ART regimen is included (red) or excluded (blue) NNRTIs, while grey lines indicate detection windows that may be within one standard deviation of the mean.

We can visually support this conclusion by examining detection windows (Fig 1b) normalized according to the model-predicted cumulative probability of viral rebound per study participant. For each study participant, we normalize the observed testing window (horizontal lines in Fig 1b) by subtracting their respective model-predicted mean time to rebound, and dividing by their respective standard deviation in time to viral rebound, as shown in Fig 6, assuming, for now, a Weibull-distributed delay. The aim of the normalization is to permit us to directly compare model predictions across study participants, showing where each individual’s “detection window” lies relative to their respective mean (normalized to zero) and standard deviation (normalized to 1) in the time to viral rebound. The model neglecting NNRTI status predicts that viral rebound outside of one standard deviation above the mean is certain, i.e., the time window between the last undetectable and first detectable viral load measurements lies entirely outside of one standard deviation above the mean, for six study participants, see Fig 6a (red lines). All six of these included NNRTIs in their pre-ATI ART regimen, and therefore the study participants who took NNRTIs (50/84) are over-represented among individuals with viral rebound delays exceeding, with certainty, one standard deviation above the mean. The model including NNRTI status, visualized in Fig 6b, shows no such over-representation.

While we cannot discern the best-fit delay distribution, we note that our parameter estimates are roughly consistent, i.e., of the same order of magnitude, across the different delay distribution models, see Table 3. Our estimates for the decay rate *k*, which is intended to correspond to the decay rate in NNRTI concentration in the body, are an order of magnitude lower than generally reported decay rates of NNRTIs measured in plasma, such as efavirenz [55]. We discuss this discrepancy in the **Discussion**.

Unless otherwise stated, for results below, we take the better-supported parameter estimates in Table 3, with a Weibull-distributed detection delay. We choose the Weibull delay distribution since it gives the lowest AIC and negative log-likelihood, given statistical equivalence of models. We attach no mechanistic significance to the Weibull distribution, although it is intriguing, since it arises as a waiting time in multi-stage models, for example in multi-stage modeling of carcinogenesis [56], and modeling of latent cell activation suggests a multi-stage process, at least in the presence of latency-reversing agents [42].

### NNRTIs induces a delay but also wide a standard deviation in time to rebound at the population level

**Fig 7:**
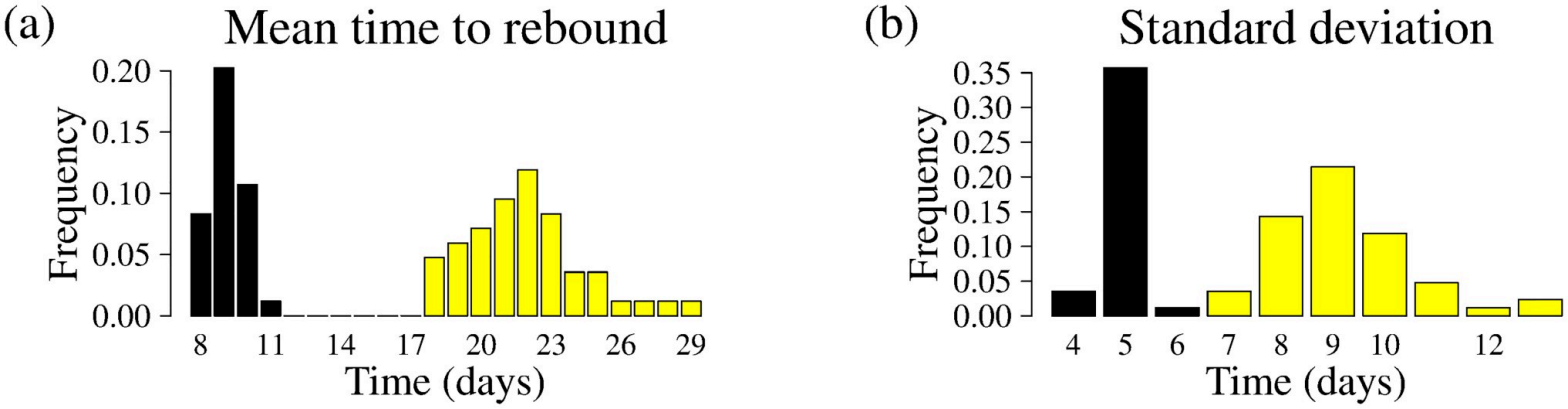
Model-predicted mean and standard deviation in time to viral rebound across the study population. Histograms show the (a) mean and (b) standard deviation in time to viral rebound across ATI study population (see **Description of data**). Black/yellow indicates absence/presence of NNRTIs in the pre-ATI ART regimen.

We show in Fig 7a predictions on the mean viral rebound time as a histogram across ATI study participants, depending on HIV CA-RNA and sorted by NNRTI status. Our model predicts that the mean time to viral rebound is delayed in individuals including NNRTIs in their pre-ATI ART regimen. This is not a surprise, as we were motivated to include the effects of NNRTIs by the observations of statistically significant delay in [2]. However, our model predictions offers additional nuance: the variation in time to rebound is also larger. Fig 7b shows a histogram of model-predicted standard deviations in time to viral rebound across the study population, again sorted by NNRTI status. The increased variability may be explained by different NNRTI drugs and individualized differences in rates of drug metabolism. We recover similar results using parameter estimates derived from other delay distribution assumptions (Table 1; not shown). The wider variation suggests that rebound times in PLWH taking NNRTIs are less predictable, and in the context of clinical trials, the inclusion of individuals on an NNRTI-based regimen may alter sample size and power calculations.

### Estimate of latent cell activation rates

We can use our parameter estimates to predict a distribution in average time to successful activation across the 84 individuals. For clarity we stress that “average” is at the individual level, since the latent reservoir is a heterogeneous population of cells. The reservoir is primarily composed of latently infected memory cells, of which there are several types, e.g., central memory, transitional memory and effector memory [44]. Investigations of latent reservoir decay after initiation of therapy show multiple decay phases [57–59], which suggest a heterogeneous population of latently infected cells with different half-lives. Further, a memory cell is only activated when it encounters its cognate antigen, e.g. a bacterial or viral peptide that it recognizes. Finally, recent evidence suggests that the reservoir is in part made up of clonal populations [60]. Therefore the activation time will vary across latently infected memory cells, depending on the rate at which an individual’s immune system is challenged with different antigens. However, if the number of cells is large, as we expect it to be in PLWH, rebound times are well described by the average.

**Fig 8:**
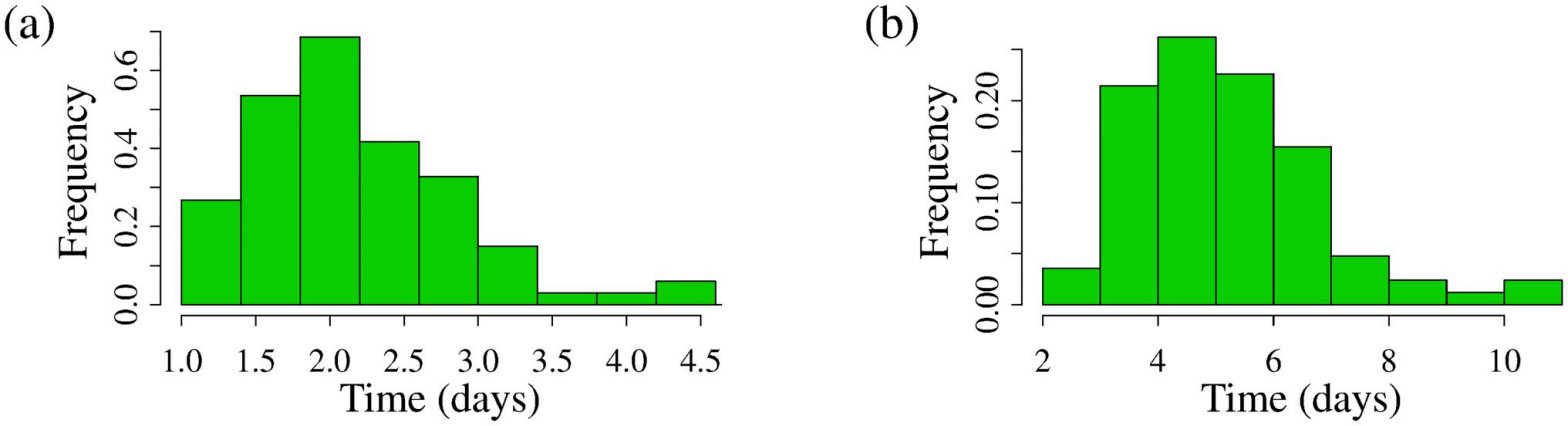
Model-predicted average frequency of recrudescence events. Histograms of model-predicted average time between successful latent cell activations, across ATI study population (see **Description of data**) assuming (a) a Weibull-distributed detection delay and (b) a fixed detection delay. The predicted population-average is a successful activation every (a) 2.2 days and (b) 5.2 days.

Our model predicts that the average frequency of successful activations in an individual is given by *as*(1 − *q*)log_10_ (CA-RNA). Fig 8 shows a histogram for the predicted time to successful activations, 1/[*as*(1 − *q*)log_10_ (CA-RNA)], across the ATI study population, assuming a Weibull-distributed (Fig 8a) or fixed (Fig 8b) detection delay.

Table 4 gives our model-predicted frequency of successful reactivation from latency, depending on the delay distribution assumption, with model-predicted means and 5th, 50th (median), and 95th percentiles. Previous modeling by Pinkevych et al. [30] estimated that the average frequency of successful reactivation from latency is about once every 6 days, and a range of 5-8 days. The result of Pinkevych et al. [30] addressed neither potential heterogeneity in the unclear latent cell kinetics post-activation [41, 42, 61], nor heterogeneity in viral growth rates as a source of rebound delays [31, 33]. In our modeling, when we neglect heterogeneity by taking a fixed delay between successful activation and infection detection, we predict an average of about 5 days (90% confidence interval 3-8 days), on par with the results of Pinkevych et al. However, when we account for that heterogeneity via alternate, i.e., non-fixed delay densities *D*(*t*), we recover a shorter average frequency of successful reactivation from latency, with successful activations occurring on average every 2-4 days (see Table 4).

**Table 4:** Model-predicted mean time to successful latent cell activation, depending on detection delay distribution assumption, across study population, with 5th, 50th (median), and 95th percentiles.

*Detection delay distribution*	Mean (days)	Percentiles (days)		
		5th percentile	50th percentile (median)	95th percentile Fixed
Fixed (*δ* − distr.) delay	5.2	3.2	5.0	8.1
Exponentially distr. delay	3.8	2.3	3.7	5.9
Gamma distr. delay	2.4	1.5	2.3	3.7
Lognormally distr. delay	2.9	1.8	2.8	4.5
Weibull distr. delay	2.2	1.3	2.1	3.4
Log-logistic distr. delay	3.3	2.0	3.2	5.1

Our result also adds nuance to previous estimates. While we estimate that the mean frequency of successful activation from latency is once every 2 (Weibull distributed detection delay) to 5 (fixed detection delay) days, we also report the population range of estimated frequencies within the 5th and 95th quantiles at most once every 3-8 days, approximately, neglecting heterogeneity and assuming a fixed detection delay, and at least 1-3 days, approximately, assuming a Weibull-distributed delay. For the purposes of further calculation, we can also use these data to estimate a population-level distribution for an individual’s average frequency of successful reactivation from which we can sample, see S2 Fig. We find that the data shown in Fig 8 is best described by a lognormal distribution, *t*_*act*_ ∼ Lognormal(*μ*, *σ*^2^) with *μ* = 0.74 (standard error 0.03) and *σ* = 0.29 (standard error 0.02) with a Weibull-distributed delay, which gives a mean average frequency of successful reactivation of once every 2.2 days with 90% confidence interval (1.3,3.4) days (S2a Fig), and *μ* = 1.61 (standard error 0.03) and *σ* = 0.30 (standard error 0.02), neglecting heterogeneity and assuming a fixed delay, which gives a mean of once every 5.2 with 90% confidence interval (3.1,8.1) days (S2b Fig).

### Clinical trial design implications

We envision the primary use of our parameter estimation to be ATI clinical trial design. Our model predicts a probability density function for a study participant’s time to viral rebound following ATI, depending on that participant’s pre-ATI log_10_(HIV CA-RNA) with level and ART regimen. We can use the predictions to plan testing intervals to capture rebound times to within study-objective specificity. We use tools from survival analysis, treating 1-*P*_*VR*_(*t*), 1-(cumulative probability of viral rebound function), as the survival function, *S*(*t*) = 1 − *P*_*VR*_(*t*).

#### Hazard rate

We can re-interpret the rate of successful latent cell activations as the “hazard rate” *h*(*t*) for the individual, i.e., the rate at which we expect viral rebound to occur, given that it has not yet happened,

$$\begin{array}{c} h\left(t\right)=-\frac{1}{S(t)}\frac{dS}{dt}=\frac{1}{1-P_{VR}\left(t\right)}\frac{d}{dt}P_{VR}\left(t\right). \end{array}$$

Assuming a fixed detection delay, we can intuit that the hazard rate is constant and equal to the successful activation rate , *h*(*t*) = *a*(1 − *q*_0_)*L*_0_ in the pre-ATI absence of NNRTIs and *h*(*t*) = *aL*_0_(1 − *q*_0_)(1 − *e*^−*kt*^) in the pre-ATI presence of NNRTIs, after the delay *t*_delay_. However, with alternate, heterogeneous detection delays, the hazard rate is not so easily obtained. An outline of the calculation is provided in the S1 Text. The hazard rate is shown in the presence or absence of NNRTIs in S3 Fig, assuming HIV CA-RNA levels across the study population (see Description of data) and a Weibull-distributed delay. We note that, although rebound is driven by a constant latent cell activation rate, assuming no NNRTIs in the pre-ART regimen, the hazard rate initially is monotonically increasing, since early viral rebound requires low-probability short detection delays, while later viral rebound reflects early successful activation with long detection delays and also late successful activation with short detection delays. Inclusion of NNRTIs in the pre-ART regimen substantially slows the increase in the hazard. We also observe that our model predicts a broad range of hazard rate across the study population, using individual HIV CA-RNA measurements to inform *L*_0_, converging after long times to between approximately 0.22 and 0.84 per day regardless of ART regimen (not depicted).

#### Testing frequency

**Fig 9:**
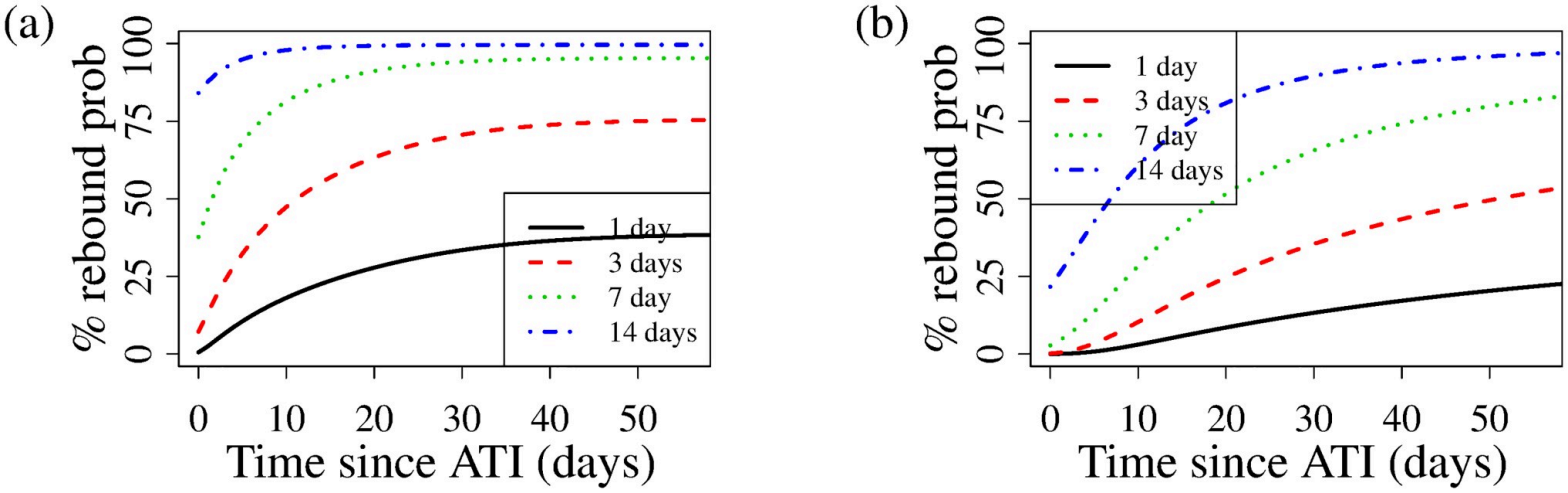
Model-recommended frequency of testing for ATI clinical trials, depending on study objectives, averaged over study population’s HIV CA-RNA levels. Probability of viral rebound given fixed testing frequencies of every 1, 3, 7, and 14 days, as a function of time since ATI for study participants whose pre-ATI ART regimen (a) excluded and (b) included NNRTIs.

While the hazard gives us insight into what to expect following ATI, we can also use our model to recommend a testing frequency aimed at capturing viral rebound for ATI studies, depending on study objectives. Fig 9a and 9b shows the probability of viral rebound per testing period as a function of time, with *t* = 0 corresponding to the start of the ATI, for different testing frequencies of every 1, 3, 7, and 14 days, for pre-ART regimens excluding (a) or including (b) NNRTIs. It is unlikely that trials will be screening for HIV CA-RNA levels prior to the ATI, so that information will likely be unknown in the course of study design; we therefore average over HIV CA-RNA levels across study participants for our predictions. The probabilities of rebound increase steadily through our 60-day rebound period, saturating to a constant, albeit more slowly in the presence of NNRTIs (Fig 9b). Therefore to ensure, for example, a probability of rebound of *no more than* 50% between tests, the model suggests a daily testing period, or every other day if NNRTIs are part of the pre-ATI ART regimen (see S4b Fig). This recommendation may be unfeasible, see Discussion below. However, our model predicts that one can relax testing frequency, early after ATI, and still achieve a fixed probability of rebound between tests (S4 Fig). To achieve, for example, approximately 25% probability of viral rebound between test dates, the first test can take place 6 days following ATI (S4a Fig), with follow-up test at day 8, then daily after that up to day 60, for a study participant with no NNRTIs in their pre-ATI ART regimen. A short discussion on how to derive these schedules is provided in the S1 Text. While the recommendation of daily testing is onerous, note that the model predicts a median time to viral rebound for such a study participant is 8 days, with 95% probability of viral rebound by day 18. For study participants whose pre-ART regimen included NNRTIs, that can be relaxed to a schedule with tests on days 15, 19, 22, 25, 28, and then every other day up to day 60. The median time to viral rebound for such a study participant is predicted to be 21 days, with 95% probability of viral rebound by day 38. Thus our model generates a recommendation permitting significantly less frequent testing near ATI, with high-frequency testing only when most of the ATI study population has already rebounded.

Our model recommends very frequent HIV viral load testing in the course of an ATI study requiring regular testing. An irregular testing schedule can remediate that challenge, but for any large trials, such irregular testing schedules may be very difficult to implement. The frequency of HIV viral load testing after treatment interruption remains controversial with studies measuring viral loads as frequently as three times per week [62] or as infrequently as once every 2-4 weeks [34, 38]. More frequent viral load monitoring after treatment interruption has potential benefits both for participant safety and maximizing the accuracy of determining HIV rebound timing, but also places a logistical burden on the participants and is not feasible for any large or medium-scale clinical trials. Thus, while our model predictions are in agreement with frequent testing recommendations, we acknowledge that logistical feasibility may dictate testing frequency.

#### Planning and resource management

**Fig 10:**
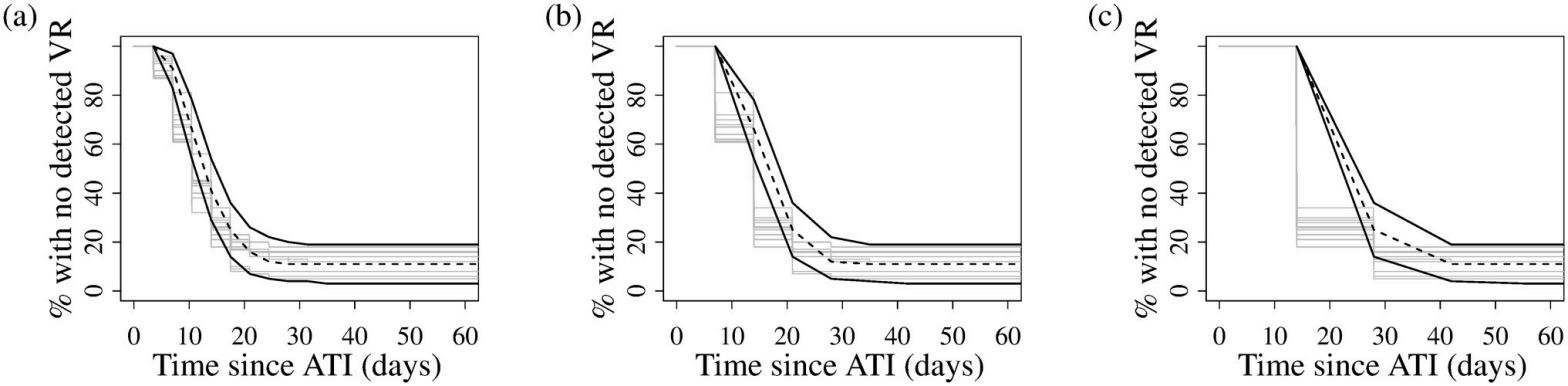
*In silico* ATI study survival curves. Ten sample survival curves for *in silico* studies with 100 study participants whose pre-ATI ART regimen included NNRTIs. Median (dashed line) and and 99% confidence interval (solid lines) computed from 10000 simulations. Survival curves describe model-predicted time to detectable viral rebound, given a post-ATI viral load testing schedule with (a) twice-weekly, (b) weekly, or (c) every two week testing.

With a prescribed testing frequency, our model can contribute to ATI clinical trial planning and resource management by predicting the course of the ATI clinical trial *in silico*. Our model and associated parametrization would represent the control population if used for a study testing, for example, interventions such as latency reversing agents or immunotherapy that may serve to extend time to viral rebound [63]. We can generate rebound times for each *in silico* study participant as follows: (i) Predict if they will rebound within ≤ 60 days, with probability 210/235, the fraction of ATI study participants who rebounded within that time period [2]. (ii) Since it is unlikely that trials will be screening for HIV CA-RNA levels prior to the ATI, that information will likely be unknown in the course of study design. Instead we can sample from the gamma distribution fit to HIV CA-RNA levels from [2], S5 Fig. (iii) We can then sample for that participant the associated viral rebound time using the cumulative probability density in Eq (2) with parameter estimates in Table 3 (taking a Weibull-distributed delay) and an assigned pre-ATI drug regimen that reflects the clinical trial enrollment criterion. We repeat this algorithm for each *in silico* ATI study participant to generate rebound times, and then use proposed testing schedules, for example twice-weekly, weekly, or every two weeks, to generate survival curves. Fig 10 shows ten sample survival curves for each of these testing schedules, assuming NNRTIs are included in the pre-ATI ART regimen, assuming for the purposes of illustration and simplicity a study of 100 individuals (see S6 Fig for the analogous figure with NNRTIs excluded from the pre-ATI ART regimen). Repeating this stochastic simulation thousands of times, we can predict, for example, the median and 99% confidence intervals on the fraction of ATI study participants who will not yet have rebound by each scheduled test time post-ATI, given in Fig 10 and S6 Fig by dashed and solid lines, respectively (see also S3 Table). These predictions may help in planning resource and personnel management, and costs, depending on study objectives.

Our model is appropriate for viral rebounds occurring up to 60 days following ATI. We hypothesize that longer-term infection control, with viral rebounds up to years after ATI, is associated with mechanisms such as immune responses excluded from this model, and which remain unclear [5, 11, 23]. Given that 210/235 total ATI study participants rebounded within 60 days [2], we anticipate our model is descriptive and appropriate for most people.

## Discussion

We have developed a simple stochastic model to predict the time to viral rebound for people living with HIV (PLWH) who undergo analytic treatment interruption (ATI). Our model predictions take the form of probability distributions in time, which can be interpreted as survival functions. Our model integrates PLWH-specific data to individualize predictions based on (1) pre-ATI ART regimen and (2) pre-ATI HIV CA-RNA, both shown to be associated with times to viral rebound [2, 3, 34]. Thus it distinguishes itself from previous studies, which have emphasized population average predictions [28–30].

In our modeling we focused on short-term viral rebound following ATI, which we restrict to ≤ 60 days. We used our model, parametrized with ATI study participant data [2], to provide a population distribution of “successful” latent cell activation rates, i.e., the rate of latent cell activations that induce viral rebound. We recover an average frequency of activations leading to viral rebound of approximately 2-4 days, depending on our assumption on the delay between activation and the increase of viremia to detectable levels. The most appropriate delay distribution cannot be resolved with existing data. Our estimate of the successful activation rate is shorter than that of Pinkevych et al. [30] who estimate an average of about 6 days between successful activations, and a range of 5-8 days [30]. Pinkevych et al. modeled viral rebound by assuming that the number of study participants controlling HIV at time *t* after ART cessation is exponentially decaying in time, and attribute that decay rate to latent cell activation. With data from treatment interruption trials, they provided the first estimates of latent cell activation rates that lead to observable viremia [30]. While our parameter estimation approach differs, the primary reason we obtain a shorter frequency of latent cell activation is that Pinkevych et al. did not explicitly address heterogeneity in the events leading to viral rebound. This heterogeneity comes from many sources including the kinetics of events that occur within a latent cell post-activation [41, 42, 61], including bursty transcription from the HIV-1 promoter that can lead to toggling between latent and pre-productive infection [64–66] and heterogeneity in subsequent viral growth rates [31, 33]. We account for heterogeneity from all sources, via the delay distribution *D*(*τ*), and thus we refine their previous estimate. However, our activation dynamics and delay distribution ignore any host factors, such as HLA allelles, that may generate inter-individual variability in time to detectable viremia. These and other host factors may become increasingly important as we strive to improve personalized predictions of viral rebound distributions.

We hypothesize that viral rebounds occurring after many months, or even years [2, 67, 68]—or not at all [5], i.e., post-treatment control—are associated with host mechanisms, such as immune responses [2, 5, 13, 23, 69]. Markers of T-cell exhaustion are associated with times to viral rebound [13]. Li et al. also noted that study participants treated early (within 6 months of exposure to HIV) showed later post-ATI viral rebound than those treated during the acute phase of infection [2]. The recent observation of rebound following ATI delayed by 7.4 months, in an individual treated within an estimated 10 days of exposure to HIV [70], is consistent with previous observations that early ART treatment is associated with delayed viral rebound timing and increased chances of post-treatment control [2, 3]. Delayed ART initiation appears to decrease the chances of sustained post-treatment viral control, potentially due to the expanding diversity of the HIV reservoir [71], immune exhaustion [72], or increasing CTL escape mutations that will diminish the effectiveness of cell-mediated immune responses [73].

As a consequence of our hypothesis, predictions of late viral rebounds may require more sophisticated models. In this present study, modeling viral rebound as a consequence of viral replication engendered by latent cell activation, we excluded data from ATI study participants whose viremia returned only after many months or years [2]. The current model acts as a necessary foundation upon which immunologic data can be incorporated when they are available to model post-treatment control. However, late viral rebounds form only a minority of dynamics following ATI, just 25 or ∼10% of 235 ATI study participants in [2], and thus our modeling, which predicts the probability of viral rebound at time *t* for PLWH following ATI in the short term, describes post-ATI dynamics in the majority of individuals. We can therefore reasonably use our modeling to aid in ATI clinical trial design, in particular determining post-ATI testing frequency, according to study objectives. If using our model for study design and implementation, we would advise reevaluation of the few individuals who achieve 60 days with no rebound with a more sophisticated approach including testing for immunological markers of HIV control [2, 5, 13, 23, 69].

From our modeling we can also identify gaps in data that may be invaluable in improving modeling insights into viral rebound and control. In creating our individualized predictions, we focused on some of the first biomarkers identified to be associated with delays in viral rebound [2]; work ongoing in identifying further covariates of control [2, 3, 10–13] will aid in further refining models of HIV rebound or control [28–30, 32, 74]. When commenting on clinical trial design we must acknowledge the practical limitations, since these studies rely on PLWH who take time out of their own lives to regularly get tested. Our data shows a median of 12 clinic visits per patient, with many making upwards of 30 clinic visits [2]. These volunteers make their contributions with the knowledge that the scientific advancements gained may not benefit them. Therefore calling for frequent testing—which from a modeling perspective would be ideal—is problematic. However we note, due to the lack of data in the first week following ATI, that we made simplifying assumptions, such as neglecting ART decay kinetics in study participants whose pre-ATI ART regimen excluded NNRTIs. Therefore we call for more regular data collection in the 1-2 weeks if possible, which may be particularly illuminating in characterizing rapid viral rebound and thus improving parameter estimation, without over-burdening clinical trialists or generous volunteers.

In our modeling we neglected the inherent heterogeneity of the latent reservoir and associated latent cell activation rates. Latently infected cells are in majority memory cells [45], each of which may be specific for a pathogen or set of pathogens. There is evidence that the reservoir is composed of clonal populations [53, 60, 75], so there may be genetically homogeneous subsets of cells, but even cells in clones may exhibit differing activation dynamics. However, in modeling viral rebound for large latent reservoir sizes, we neglect this heterogeneity in favor of the mean activation rate. Heterogeneity in activation rate becomes important as latent reservoir sizes gets small, and we move towards elimination, but that is not the focus of this present study.

The latent cell activation rate, *a*, is part of the recrudescence rate, *as*(1 − *q*_0_), which we estimate from the data and which we assume to be the same for all individuals. Note that we can only estimate the parameter combination *as*(1 − *q*_0_), and not *a* itself. Future modeling efforts may aim to rectify this possibly by including additional data, such as direct estimates of the pre-ATI viral reservoir size and viral growth rates and viral set points post-ATI for each study participant.

In accounting for the delay in viral rebound observed in Li et al. (2016) associated with inclusion of NNRTIs in the pre-ATI ART regimen [2], we assumed that the probability that latent cell activation induces viral rebound decays expontentially to *q*_0_ at rate *k*. Our estimate for *k*, which should account for the rate of drug decay post-ATI, was at least order of magnitude lower than the mean NNRTI concentration decay rates in plasma [55]. One explanation is that most T-cells and latently infected cells reside in lymphatic tissues [76, 77]. The pharmacokinetics and pharmacodynamics in the lymphatic tissues are not clear, although there is evidence that drug penetration is lower [78, 79]; commensurately, drug clearance may also be slower. Since our model crudely treats the whole body as homogeneous, the expression for decaying effectiveness of the drug must average the dynamics in different tissues, potentially explaining the inconsistencies.

It is also interesting to note that the NNRTI efavirenz’s clearance is dependent on CYP450 2B6 gene polymorphism and there are certain polymorphisms that increase plasma half life such that drug levels above the 95% inhibitory concentration maybe present for > 21 days after treatment interruption [48]. However, we acknowledge that our estimates of the rate at which drug loses effectiveness will need to be further refined and validated. In future studies we will attempt to refine *q*(*t*) and more carefully address its variability, potentially, by considering specific drug pharmacokinetic/pharmacodynamics in tissues, when available, and data on viral dynamics post-rebound to inform the reproductive ratio in absence of ART. It is also possible that ART decay may occur in a biphasic manner with our estimate of *k* reflecting the terminal elimination phase. Additional pharmacokinetic studies are needed to explore this possibility.

We also simplified our model by taking a constant reservoir size, neglecting factors contributing to long-term reservoir decay such as latent cell death and proliferation [45]. Preceding viral rebound, we anticipate that the latent reservoir would continue to decay at on-therapy rates [43, 80] resulting from these dynamics. But the reservoir is typically large and its decay is slow, with a 44 month half-life on average [43, 80], so in our 60-day rebound period, the average reservoir size would decrease by less than 3%. Thus our constant reservoir size assumption is reasonable. We can extend our simple model to include latent cell proliferation and death, derived in the S1 Text. Intriguingly, the resulting expression for probability of viral rebound gives the natural activation rate *a* as, in principle, an identifiable parameter, in contrast to our simple model, for which only the successful latent cell activation rate, (1 − *q*)*aL*_0_, is identifiable. Unfortunately, to disentangle *a* from other parameters in the extended model, we require more refined data, as discussed based on the mathematics in the S1 Text. Such data may be difficult to obtain; as it is, the data from Li et al. [2] which we employ is the most extensive and well-curated ATI study data currently available.

In integrating study participant data into our viral rebound model, we made the assumption that log_10_(cell-associated HIV RNA) is proportional to the size of the replication-competent portion of the latent reservoir, for which we currently have no direct methods to measure [46]. We were motivated by Li et al., who showed a negative correlation between HIV CA-RNA and time to detectable viremia [2]. Li et al. also noted a negative correlation between pre-ATI viral load, measured using single copy assays, and time to detectable viremia [2]. Since on-therapy viremia may be associated with rounds of replication resulting from latent cell activations [24], pre-ATI viral load may be a better measure of the replication-competent portion of the latent reservoir. It also may not: if ART efficacy is near 100%, there may be only very limited rounds of replication. In that case, on-therapy viremia would represent primarily virus released from activated latently infected cells, and would not be a good measure of replication competence, although the higher the fraction of cells releasing HIV, the higher the probability that the population will include cells releasing replication competent virus [53]. Regardless, we did not incorporate this observation into the current model because of the paucity of data (41/235 ATI study participants with viral load measured above the level of detection), but we acknowledge that the pre-ATI viral load may be a better predictor of replication competent latent reservoir size.

Our simple model predictions suggest that any given treated HIV^+^ individual who will undergo what we term ‘short-term rebound’ is capable of reproducing almost the full amount of variability in rebound times seen across the 84 study participants under consideration (c.f. Figs 5b, 5c and 7), with some finite probability, which varies across study participants. For example, the model predicts that the probability of rebound within two weeks of ATI across study participants ranges from 11% to 90%, depending on the study participant, and the probability of rebound after 6 weeks ranges from 10^−5^% to 15% (Fig 5b and 5c). Predicted mean times to viral rebound vary from days, without NNRTIs in the pre-ATI ART regimen, to weeks with NNRTIs (Fig 7). One interpretation is that the observed variability in rebound times is driven most strongly by stochasticity in the delay between successful activation and viral detection, and in the time to reactivation, with an individual’s level of HIV CA-RNA influencing the time to viral detection (Fig 3b). However, we suspect that the similarity in predicted viral rebound time probability density functions in the absence of NNRTIs, in particular Fig 5a and 5b, also importantly reflects the uncertainty in the data with respect to actual rebound times; recall that the median time between the last undetectable viral load measurement and first detectable viral load measurement is 20 days (mean 18 days). While parameter estimation with more frequent observations would improve predictions and resolve this question, such data may only be obtained with difficulty, as again the data we employ here derives from the most extensive ATI study data currently available. We therefore advise mindfulness of the uncertainty as modeling of viral rebound advances.

While we acknowledge many limitations, our simple model, parametrized with ATI study participant data, offers individualized predictions of time-to-viral rebound following ATI. Our results offer insight into latent cell activation dynamics, can inform future modeling and predictive work, and can be used to inform testing periods in ATI clinical trial design. This study represents first steps towards a model that can make accurate predictions of a person living with HIV (PLWH)’s rebound time distribution based on personal characteristics, and help identify PLWH with expected long viral rebound delays.

## Supporting information

S1 Text Supporting calculations. (PDF)

S1 Table Parameter estimation for model 1. Parameter estimates for main text model (1) with 95% confidence intervals indicated parenthetically, making no distinction between participants based on pre-ATI ART regimen.
(PDF)

S2 Table Parameter estimation for model 2. Parameter estimates for main text model (2) with 95% confidence intervals indicated parenthetically, distinguishing study participants based on pre-ATI ART regimen.
(PDF)

S3 Table Clinical trial predictions. Median and 99% confidence interval in the fraction of ATI study participants showing no rebound by each test time post-ATI, computed over 10000 simulations. See text for algorithm generating the survival curves from which these were derived. For the purposes of illustration we assume 100 study participants.
(PDF)

S1 Fig Model-predicted probability density functions for delay *D*(*t*). Model-predicted probability density functions for delay between activation that induces rebound and detectable viremia, *D*(*t*), parameters estimated for main text Eq (2). Probability density formulas are provided in main text Table 1 and parameter estimates for the probability densities are given in Table.
(PDF)

S2 Fig Histograms of model-predicted average time between successful latent cell activations, across ATI study population. Histograms of model-predicted average time between successful latent cell activations, across ATI study population (see main text, **Description of data**), assuming (a) a Weibull-distributed detection delay and (b) a fixed detection delay, with lognormal distribution fit shown in red. The lognormal distribution fits is best when compared to some stylized fits (tested exponential, Weibull, and Burr distributions, ΔAIC > 4.5) but statistically indistinguishable from others (tested gamma and log-logistic distributions, 2 < ΔAIC < 3). The population-average is predicted to be a successful activation every (a) 2.2 days, (b) 5.2 days.
(PDF)

S3 Fig Predicted rate of viral rebound (hazard) depending on pre-ATI drug regimen. Areas indicate span of predicted rebound rates depending on pre-ATI HIV CA-RNA level across all data, with green indicating data from study participants who included NNRTIs in their pre-ATI ART regimen, and blue indicating study participants who did not. The solid/dashed lines indicate the rebound rates assuming median HIV CA-RNA levels for each group.
(PDF)

S4 Fig Model-recommended frequency of testing for ATI clinical trials. Model-recommended frequency of testing for ATI clinical trials, depending on study objectives, averaged over study participant HIV CA-RNA levels. Next-test time given a fixed, desired, probability of viral rebound, as a function of time since ATI, for study participants whose pre-ATI ART regimen (a) excluded and (b) included NNRTIs.
(PDF)

S5 Fig Histograms of log_10_(HIV CA RNA) levels measured across study participants. Histograms of log_10_(HIV CA RNA) levels from [2] (see main text, **Description of data**), with gamma distribution fit. The gamma distribution fits is best when compared to some stylized fits (tested Weibull, log-normal, log-logistic, and Burr distribution) yielding the lowest log-likelihood. Since all tested distributions have the same number of parameters, we have no need to call on the AIC.
(PDF)

S6 Fig *In silico* ATI study survival curves. Ten sample survival curves for *in silico* studies with 100 study participants whose pre-ATI ART regimen excluded NNRTIs. Median (dashed line) and and 99% confidence interval (solid lines) computed from 10000 simulations. Survival curves describe model-predicted time to detectable viral rebound, given a post-ATI viral load testing schedule with (a) twice-weekly, (b) weekly, or (c) every two week testing.
(PDF)
