# Supplementary material for: Predictions of time to HIV viral rebound following ART suspension that incorporate personal biomarkers
Source: PLoS Comput Biol. 2019 Jul 24;15(7):e1007229. doi: 10.1371/journal.pcbi.1007229 (PMC6682162; doi:10.1371/journal.pcbi.1007229)
Supplement: S1 Text — (PDF) [file pcbi.1007229.s001.pdf]

Predictions of time to HIV viral rebound following ART suspension that incorporate personal biomarkers

# - Supporting information -

Jessica M. Conway, Alan S. Perelson, and Jonathan Z. Li

A Derivations of the probability of viral rebound

Here we outline the derivation of the probability of viral rebound, main text eqs. (1) and (2), following derivations in [1–3]. We hypothesize that short-term viral rebound is associated with latent cell activation, and that an activation fails to induce viral rebound with probability q. In the following we first derive the probability of viral rebound, main text eqs. (1) and (2), assuming that the latent reservoir size is constant L_0, which is used for the main results of this paper. We then derive the analogous probability of viral rebound assuming dynamics on the latent reservoir, specifically that latently infected cells die and can proliferate [4], and that in the presence of low viral loads on par with treated infection, the latent reservoir decays with a half-life $t_{1/2} = 44$ months [5, 6].

A.1 Model of viral rebound assuming constant latent reservoir size

For short term viral rebounds (≤ 60 weeks), assume that the latent reservoir size is constant L_0, cells are activated at average rate a, and that an activated cell successfully induces viral rebound with probability $1-q$. Thus the successful activation rate is $aL_0(1-q)$. To compute the probability of viral rebound at time t, we use the probability density of no successful latent cell activations at time t. Let $L(t)$ and $A(t)$ be random variables giving the number of latently infected cells and successful activations at time t. We define

$$P_{\ell,n;\ell_0,n_0}(t,\tau) = P(L(t) = \ell, A(t) = n | L(\tau) = \ell_0, A(\tau) = n_0)$$

as the probability that there are ℓ latently infected cells and n activations at time t given ℓ_0 latently infected cells and n_0 activations at initial time τ. We derive the probability of viral rebound for generic initial conditions ℓ_0 and n_0 and then impose our initial conditions, $\ell_0 = L_0$ and $n_0 = 0$ to derive the final answer. Following methods described in [1–3] we derive the backwards Chapman Kolmogorov equations for

$P_{\ell,n;\ell_0,n_0}(t,\tau)$,

$$\frac{\partial P_{\ell,n;\ell_0,n_0}}{\partial \tau} = -A(\tau)\ell_0\left(P_{\ell,n;\ell_0,n_0+1} - P_{\ell,n;\ell_0,n_0}\right)$$

with terminal condition $P_{\ell,n;\ell_0,n_0}(t,t) = \delta_{\ell,\ell_0}\delta_{n,n_0}$, for $\delta_{i,j}$ the Kronecker-Delta function. Note that this ODE run backwards in time, with τ decreasing from t down to 0. We have defined the successful activation rate $A(\tau)$, depending on the pre-ATI drug regimen,

$$A(\tau) = \begin{cases} a(1-q_0), & \text{in the absence of NNRTIs} \\ a\left(1-q_0\right)\left(1-e^{-k\tau}\right), & \text{in the presence of NNRTIs,} \end{cases} \tag{A1}$$

where we assume for simplicity that all drugs instantly wash out at the time of ATI for regimes that do not contain NNRTIs, and wash out exponentially with rate k for NNRTI-containing regimes.

Note that realistically, non-NNRTI therapy must also have some short-term effect following ATI. However non-NNRTI ART drugs typically have short half lives [7] and our data in the first week following ATI is sparse, with a first detectable viral load in study participants not taking NNRTIs only at day 6, and 4 participants with measured detectable viral load at day 10, all with last undetectable viral load measurement at day 0. Therefore the rapid decay would be difficult, if not impossible, to detect, and for simplicity only we assume instant washout.

Defining the probability generating function $G_{\ell_0,n_0}(x,y;t,\tau) = \sum_{\ell,n} P_{\ell,n;\ell_0,n_0}(t,\tau)x^{\ell}y^{n}$ and exploiting the branching property $G_{\ell_0,n_0}(x,y;t,\tau) = G_1^{\ell_0}G_2^{n_0}$, where $G_1 \equiv G_{1,0}$ and $G_2 \equiv G_{0,1}$, the ordinary differential equations for the generating function are

$$\begin{aligned} \frac{\partial G_1}{\partial \tau} &= -A(\tau)G_1(G_2-1) \\ \frac{\partial G_2}{\partial t} &= 0 \end{aligned}$$

with terminal conditions $G_1(x,y;t,t) = x$ and $G_2(x,y;t,t) = y$. Solving these, we recover $G_1(x,y;t) \equiv G_1(x,y;t,0) = xe^{(y-1)\int_0^t A(\tau)\,d\tau}$, $G_2 = y$.

Now we apply our initial conditions. Since the fixed latent reservoir size is L_0 and we assume no activations so far, i.e., $n_0 = 0$, our probability generating function $G \equiv G_{L_0,0}$ is

$$G(x,y;t) = x^{L_0}e^{(y-1)L_0\int_0^t A(\tau)\,d\tau}.$$

We want the probability of no successful activations at time t, $P_{L_0,0;L_0,0}(t) \equiv G(1,0;t) = e^{-L_0 \int_0^t A(\tau)\,d\tau}$. Then the cumulative probability of a successful activation at time t is $1 - e^{-L_0 \int_0^t A(\tau)\,d\tau}$. Finally, since we assume that detectable viremia arises a time s from successful activation with probability density function $f(s)$ (see main text), the probability of viral rebound at time t is

$$P_{VR}(t) = \int_0^t \left(1 - e^{-L_0 \int_0^{t-s} A(\tau)\,d\tau}\right) f(s)\,ds. \tag{A2}$$

With provided $A(\tau)$, eq. (A1),

$$P_{VR}(t) = \begin{cases} \int_0^t \left(1 - e^{-(1-q_0)aL_0(t-\tau)}\right) f(\tau)\,d\tau & \text{in the absence of NNRTIs} \\ \int_0^t \left\{1 - \exp\left[-\frac{aL_0(1-q_0)(\exp(-k(t-\tau))-1+k(t-\tau)}{k}\right]\right\} f(\tau)\,d\tau, & \text{in the presence of NNRTIs} \end{cases} \tag{A3}$$

A.2 Modeling latent reservoir dynamics

In the main text, we briefly discuss a model analogous to eq. (A3) with dynamics on the latent reservoir: instead of a constant latent reservoir size, we assume that in addition to latent cell activation, latently infected cells can proliferate at rate ρ and die at rate μ. Latent cell activation at rate a now also causes cell loss. As in Sec. A.1 we assume that activations fail to induce viral rebound at probability q. Here we derive those equations.

A.2.1 Probability of viral rebound at time t

As in Sec. A.1 we formulate our model using multi-type, continuous time branching processes, defining $P_{\ell,n;\ell_0,n_0}(t) = P(L(t) = \ell, A(t) = n | L(0) = \ell_0, A(0) = n_0)$ as the probability of ℓ latently infected cells and n activations at time t, assuming that there were ℓ_0 and n_0 latently infected cells and activations, respectively, at time 0. As before, to compute the probability of viral rebound at time t, we first compute the cumulative probability of a first successful activation at time t. Now accounting for additional latent reservoir dynamics, the forwards Chapman Kolmogorov differential equation, also called master equation, that describes these dynamics is

$$\begin{aligned} \frac{dP_{\ell,n;\ell_0,n_0}}{dt} &= \rho(\ell-1)P_{\ell-1,n;\ell_0,n_0} + a(1-q)(\ell+1)P_{\ell+1,n-1;\ell_0,n_0} + (aq+\mu)(\ell+1)P_{\ell+1,n;\ell_0,n_0} \\ &\quad -(\rho+a+\mu)\ell P_{\ell,n;\ell_0,n_0} \end{aligned}$$

with initial condition $P_{\ell,n;\ell_0,n_0}(0) = \delta_{\ell,\ell_0}\delta_{n,n_0}$. For ease of analysis, we use the associated backwards Chapman Kolmogorov differential equation,

$$\frac{dP_{\ell,n;\ell_0,n_0}}{dt} = \ell_0\left(\rho P_{\ell,n;\ell_0+1,n_0} + a(1-q)P_{\ell,n;\ell_0-1,n_0+1} + aqP_{\ell,n;\ell_0-1,n_0} + \mu P_{\ell,n;\ell_0-1,n_0} - (\rho+a+\mu)P_{\ell,n;\ell_0,n_0}\right)$$

again with initial condition $P_{\ell,n;\ell_0,n_0}(0) = \delta_{\ell,\ell_0}\delta_{n,n_0}$. Here we neglect the case where the probability of failed activation is time-dependent, i.e., that there are NNRTIs present, and therefore can treat this backwards Chapman Kolmogorov differential equation as forward in time. Defining the probability generating function $G_{\ell_0,n_0}(x,y;t) = \sum_{\ell,n} P_{\ell,n;\ell_0,n_0} x^\ell y^n$ and exploiting the branching property $G_{\ell_0,n_0}(x,y;t) = G_1^{\ell_0} G_2^{n_0}$, where $G_1 \equiv G_{1,0}$ and $G_2 \equiv G_{0,1}$, the ordinary differential equations for the generating function are

$$\begin{aligned}\frac{dG_1}{dt} &= (\mu - \rho G_1)(1-G_1) + a(1-q)G_2 + aq - aG_1\\ \frac{dG_2}{dt} &= 0\end{aligned}$$

with initial condition $G_1(0) = x$ and $G_2(0) = y$. Solving these, we recover $G_2 \equiv y$ and

$$G_1(x,y;t) = \frac{(2(a+\mu) - 2a(1-y)(1-q) - x(\rho+a+\mu))\tanh[\phi t/2] + x\phi}{(-2\rho x + \rho + a + \mu)\tanh[\phi t/2] + \phi},$$

where $\phi = \sqrt{(\rho - a - \mu)^2 + 4a\rho(1-y)(1-q)}$. Since the initial latent reservoir size is L_0 and we assume no activations at time 0, i.e., $n_0 = 0$, our probability generating function $G \equiv G_{L_0,0}$ is

$$G(x,y;t) = \left(\frac{(2(a+\mu) - 2a(1-y)(1-q) - x(\rho+a+\mu))\tanh[\phi t/2] + x\phi}{(-2\rho x + \rho + a + \mu)\tanh[\phi t/2] + \phi}\right)^{L_0}$$

To get to the cumulative probability of viral rebound at time t, we use the probability of no successful activations at time t, $P_{L_0,0;L_0,0}(t) \equiv G(1,0;t)$, and the cumulative probability of a successful activation at time t is $1 - G(1,0;t)$. Finally, since we assume that detectable viremia arises a time s from successful activation with probability density function $D(s)$ (see main text), the probability of viral rebound at time t is $P_{VR}(t) = \int_0^t (1 - G(1,0;t-s))D(s)\,ds \Rightarrow$

$$P_{VR}(t) = \int_0^t \left[1 - \left(\frac{(a+\mu-2a(1-q)-\rho)\tanh[\phi(t-s)/2] + \phi}{(-\rho+a+\mu)\tanh[\phi(t-s)/2] + \phi}\right)^{L_0}\right] D(s)\,ds \qquad \text{(A4)}$$

where $\phi = \sqrt{(\rho - a - \mu)^2 + 4a\rho(1-q)}$.

A.2.2 Probability of viral rebound as $t \to \infty$

In absence of replenishment by high levels of viral replication, the latent reservoir decays with a half-life $t_{1/2} \approx 44$ months [5, 6]. Therefore, the latent reservoir can be eliminated in finite time, and viral rebound is no longer guaranteed as $t \to \infty$. The probability of viral rebound is then $P_{VR}^{\infty} = \lim_{t\to\infty} P_{VR}(t)$,

$$P_{VR}^{\infty} = 1 - \left(\frac{-\rho + a + \mu - 2a(1-q) + \phi}{-\rho + a + \mu + \phi} \right)^{L_0}.$$

Then taking the net latent reservoir decay rate $\rho - a - \mu = -\ln(2)/t_{1/2}$ where $t_{1/2}$ is the latent reservoir half-life, we can write the the proliferation rate ρ as $\rho = a + \mu - \ln(2)/t_{1/2}$.

$$P_{VR}^{\infty} = 1 - \left(1 - \frac{2a(1-q))}{(\ln 2/t_{1/2}) + \phi} \right)^{L_0}. \tag{A5}$$

where $\phi = \sqrt{\left(\ln 2/t_{1/2}\right)^2 + 4a\left(a + \mu - \ln 2/t_{1/2}\right)(1-q)}$.

A.2.3 Parameter identifiability

In the main text we discuss how, for the purposes of estimating parameters in our expression of probability of viral rebound at time t assuming a constant latent reservoir size, eq. (A3), $a(1-q_0)L_0$ form a non-identifiable parameter combination. When we account for dynamics on the latent reservoir in computing the cumulative probability of viral rebound at time t, eq. (A4), the natural latent cell activation rate a seems to be separately identifiable. Direct estimation of a would certainly be a boon, and enlightening in modeling studies aiming to eliminate the latent reservoir. However, a is likely small. If we take a small and take the Taylor series of $P_{VR}(t)$ in eq. (A4), to first order,

$$P_{VR}(t) \approx \frac{aL_0(1-q)\left(1 - 2^{-(t-s)/t_{1/2}}\right)}{\ln(2)/t_{1/2}}$$

(taking, for the purpose of expediency, a $\delta-$distributed delay $D(t) = \delta(t-s)$). Similarly, for small a, the limiting probability of viral rebound $P_{VR}^{\infty} \approx aL_0(1-q)t_{1/2}/\ln(2)$. Therefore, to leading order, the identifiability issue remains. From this observation we conclude that a model including latent reservoir dynamics

may permit identification of the natural activation rate a. However, disentangling a from $(1-q)$ would require a powerful and refined data set, which would be difficult to generate or acquire; the data set that we are using, from Li et al. (2016) [8] is already the most impressive and extensive of its kind.

B On $q(t)$, the probability of extinction in the presence of NNRTIs

In the presence of NNRTIs, we take the time-dependent probability that an activated latently infected cell induces viral rebound as $1-q(t)$, where

$$q(t) = q_0 + (1-q_0)e^{-kt}. \tag{B1}$$

q is the probability that the lineage of infected cells and virus induced by the activation of a latently infected cell ultimately goes extinct. This functional form is a highly simplified and crude approximation, but with some justification, which we offer here.

In the main text, we present a viral dynamics model that does not follow the standard viral dynamics paradigm. That is, we do not assume that a latently infected cell is a effectively a productively infected cell, which then follows standard viral dynamics [9, 10]. A discussion of why we avoid that common assumption can be found in the main text. In brief, (1) In vitro data suggests that when you activate a latently infected cell in vitro, virus produced is less by an order of magnitude at least relative to productively infected cells [11]. (2) Following the application of latency reversing agents, latently infected cells dynamics do not conform to observed dynamics of productively infected cells [12]. (3) Culture work suggests that latently infected cells may also divide before they get fully activated, before producing virus [11]. (4) Finally, the standard viral dynamics assumes proximity of target cells to infect, which may not be consistent with dynamics following latent cell activation in vivo.

These dynamics would generally serve to reduce the probability that latent cell activation induces viral rebound, relative to the assumption that a latently infected cell is effectively a productively infected cell. We can therefore take as an upper bound for the probability of viral rebound $1-q$ the probability that a single productively infected cell induces viral rebound.

We assume that viral dynamics for productively infected cells and virus follow the stochastic analogue of the viral dynamics model with a constant number of target cells [1, 13], a fair assumption preceding viral

rebound, since target cells are only depleted when viral loads are high. Then the probability that the lineage created by a single infected cell goes extinct with probability

$$p_{\text{ext}} = \min\left(\frac{1}{R}, 1\right),$$

where R is the reproductive ratio given an approximately constant target cell count preceding viral rebound. Note that we assume continuous viral production from infected cells here; if we instead assumed viral production occurs in a burst, $p_{\text{ext}} = \min\left(p^N, 1\right)$ where p is the root of $R(1-p^N) = N(1-p)$ [1,13].

The reproductive ratio R, the number of new cell infections engendered by a single infected cell given the target cell population, must decay with drug concentration. The time for this decay to happen depends on the maximal drug levels at the time of ATI, C_{max}, and the dose-response curve describing the fractional inhibition in viral replication as a function of the drug levels, which is often described by a Hill function with parameters IC_{50} and m, the Hill coefficient that determines the steepness of the dose response curve, and the baseline reproductive ratio, R_{00}, in absence of drugs, again preceding viral rebound, respectively. For a single drug, R would increase over time following [14]:

$$R(t) = \frac{R_{00}}{(1 + (\text{C}_{\text{max}} e^{-t\ln 2/t_{1/2}}/\text{IC}_{50})^m)}$$

assuming drug levels decline completely exponentially with decay rate $m\ln 2/t_{1/2}$, where $t_{1/2}$ is the drug half-life. The expression for $R(t)$ would be more complicated for multiple drugs. However, most non-NNRTI drugs have half lives on the order of hours, up to at most a day [7], and our data only shows viral rebound detected in study participants after 6 days post-ATI (and, for study participants whose NNRTI regimen included NNRTIs, only one had detected viral rebound before day 12). Therefore we cannot discern from the data interaction with other drugs and focus on the de facto monotherapy in the "NNRTI tail" [15].

We can therefore support our crude approximation for the probability of extinction $q(t)$ from the standard viral dynamics model and pharmacodynamics modeling,

$$p_{\text{ext}}(t) \quad = \quad \min\left(\frac{1}{R_0(t)}, 1\right) = \min\left(\frac{1}{R_{00}}\left(1 + \left(\frac{C_{\text{max}}}{IC_{50}}\right)^m \exp\left(-m\frac{\ln 2}{t_{1/2}}t\right)\right), 1\right).$$

This expression suggests that there may be some period of time, while $R_0(t) < 1$, where extinction remains

guaranteed. Then assuming that $1/R_0(\tau) = 1$ we can write

$$p_{\mathrm{ext}}(t) = \begin{cases} 1, & 0 \le t < \tau \\ q_0 + (1-q_0)e^{-k(t-\tau)}, & t \ge \tau \end{cases},$$

taking $q_0 = 1/R_{00}$, the probability of extinction in absence of therapy, $k = m\ln(2)/t_{1/2}$, and $\tau = \frac{1}{k}\ln\left\{(C_{\max}/IC_{50})^m / (R_{00}-1)\right\}$.

Note that in our expression, eq. (B1), $q(t) = p_{\mathrm{ext}}(t)$, with neglected τ. This choice was made out of necessity, due to the limited early sampling in the data: if we take it to be a fixed parameter with some uncertainty, it must be less than 6 to permit rebound at day 6, and with only limited sampling in the first week following ATI, it is difficult to discern. Indeed our attempts to estimate τ either did not converge or led to estimates so small we suspect identifiability issues (for the reader's information, AIC of fits with the estimated very smallτ exceed those for fits in the main text). We therefore only focus on the dynamics in the "NNRTI tail" [15], modeled with $q(t) = q_0 + (1-q_0)e^{-kt}$.

Since $q(t)$ is derived from a pharmacodynamics model, we may well ask what $p_{\mathrm{ext}}(t)$ is using parameter estimates from PK/PD of NNRTI drugs, for example for efavirenz. In that case, $\mathrm{IC}_{50} \approx 5\,\mathrm{nM}$, $m \approx 1.7$, $t_{1/2} \approx 45\,\mathrm{hrs}$, $C_{\max} \approx 13\,\mu\mathrm{M}$ [16, 17]. Then $k \approx 0.6$ per day, and taking the broad range $R_{00} = 1.1 - 20$, $q_0 \approx 0.05 - 0.91$, and $\tau \approx 17 - 25$ days.

The implication of τ is that, neglecting the possibility of emergent drug resistance, no study participant can rebound within τ days of ATI, but we almost a quarter of our study participants have confirmed detectable viremia by 17 days post-ATI. And our estimated decay rate k is an order of magnitude larger than our estimates (see main text). Though estimates of k are roughly consistent with the observation of drug levels above the 95% inhibitory concentration present for a median of 6-14 days after treatment interruption [22], there is a discrepancy.

We note that the estimates [16] rely on measurements in the blood, while most T-cells and latently infected cells reside in lymphatic tissues [18, 19]. PK/PD dynamics in the lymphatic tissues are not clear, although there is evidence that drug penetration is lower [20, 21]. Perhaps drug clearance is also slower. Since our model crudely treats the whole body as homogeneous, the expression for decaying effectiveness of the drug must average the dynamics in different tissues, potentially explaining the inconsistencies. Alternatively, while in our modeling, accounting for NNRTI in the pre-ATI ART regimen does explain the

data better than when it is neglected, it is a rough representation of dynamics. Perhaps a more sophisticated model, supported by data and empirical observations, which we currently don't have, would be more consistent with established PK/PD dynamics.

C Derivation of the hazard rate

The primary use of our parameter estimation that we envision is ATI clinical trial design. Specifically, we can use the model-predicted probability density function for a study participant's time to viral rebound following ATI, depending on that participant's pre-ATI $\log_{10}$(HIV CA-RNA) level and ART regimen, to create testing intervals to capture rebound times within study-objective specificity. To this end, we use tools from survival analysis, treating 1-$P_{VR}(t)$, 1-(cumulative probability of viral rebound function), as the survival function, $S(t) = 1 - P_{VR}(t)$. In the main text we discuss the "hazard rate" $h(t)$ for the individual, i.e., the rate at which we expect viral rebound to occur, given that it has not yet. If we assume a fixed delay between successful latent cell activation and detectable viremia, the hazard rate $h(t)$ is intuitively, after the delay, equal to the rate of successful latent cell activations. If, however, we assume a general probability distribution function for the delay, $f(t)$ (cf. main text Table 1), the hazard rate is not so intuitive direct and must be derived mathematically,

$$h(t) = -\frac{1}{S(t)}\frac{dS}{dt} = \frac{1}{1 - P_{VR}(t)}\frac{d}{dt}P_{VR}(t).$$

We have derived probability of viral rebound assuming delay probability density $f(t)$, given in eq. (A2), $P_{VR}(t) = \int_0^t \left(1 - e^{-L_0 \int_0^{t-\tau} A(s)\,ds}\right) f(\tau)\,d\tau$, which we can re-write as $P_{VR}(t) = F(t) - \int_0^t e^{-L_0 \int_0^{t-\tau} A(s)\,ds} f(\tau)\,d\tau$, where $F(t)$ is the cumulative density function associated with the probability density function $F(t)$. Then we can calculate the hazard rate as

$$\begin{aligned} h(t) &= \frac{1}{1 - P_{VR}(t)}\frac{d}{dt}P_{VR}(t) \\ &= \frac{\int_0^t A(t-\tau) L_0 e^{-L_0 \int_0^{t-\tau} A(s)\,ds} f(\tau)\,d\tau}{1 - F(t) + \int_0^t e^{-L_0 \int_0^{t-\tau} A(s)\,ds} f(\tau)\,d\tau} \end{aligned}$$

where $A(\tau)$ is the rate of successful activations, in the presence or absence of NNRTIs, given by eq. (A1). Accounting for this presence/absence we recover

$$h(t) = \begin{cases} \frac{aL_0(1-q_0)\int_0^t e^{-aL_0(1-q_0)(t-\tau)} f(\tau)\,d\tau}{1-\int_0^t \left(1-e^{-aL_0(1-q_0)(t-\tau)}\right) f(\tau)\,d\tau}, & \text{in the absence of NNRTIs} \\ \frac{aL_0(1-q_0)\int_0^t \left(1-e^{-k(t-\tau)}\right) e^{-aL_0(1-q_0)(\exp(-k(t-\tau))-1+k(t-\tau))/k} f(\tau)\,d\tau}{1-\int_0^t \left(1-e^{-aL_0(1-q_0)(\exp(-k(t-\tau))-1+k(t-\tau))/k}\right) f(\tau)\,d\tau}, & \text{in the presence of NNRTIs.} \end{cases}$$

In the main text we take for $f(t)$ and $F(t)$ the Weibull probability and cumulative density functions, respectively. Note that in both cases, the hazard rate converges to $aL_0(1-q_0)$, i.e., $\lim_{t\to\infty} h(t) = aL_0(1-q)$. Though as we see in main text Fig. 9 that, for our parameter estimates, convergence takes months.

D Testing window calculation and discussion

In the main text, Fig. 9, we provide model predictions for required frequency of testing for ATI clinical trials, depending on study objectives, averaged over HIV CA-RNA levels across study participants. Here we provide details of the associated calculation, and a visual overview of how to estimate a testing schedule from Fig. S4c,d keeping the inter-test probability of viral rebound approximately constant.

D.1 Calculation details

We want to compute the probability of viral rebound by time t_1, given no viral rebound by time t_0, for $t_1 > t_0$. Recall that $P_{VR}(t)$ is the cumulative probability of viral rebound by time t. Assume that $p_{VR}(t)$ is the associated probability density, $p_{VR}(t) = P'_{VR}(t)$. The probability density of viral rebound at time $t > t_0$, given no rebound by time t, is

$$\tilde{p}_{VR}(t) = \begin{cases} 0, & t \leq t_0 \\ \frac{p_{VR}(t)}{\int_{t_0}^{\infty} p_{VR}(t)\,dt}, & t > t_0 \end{cases} = \begin{cases} 0, & t \leq t_0 \\ \frac{p_{VR}(t)}{1-P_{VR}(t_0)}, & t > t_0 \end{cases}$$

Essentially we are re-normalizing the probability density so the area under the curve is 1 for $t > t_0$. Then the probability of viral rebound in $(t_0, t_1]$, given that there has been no viral rebound by time t_0, is given by

the integral

$$
\begin{aligned}
\text{Prob}(\text{rebound in } (t_0, t_1] | \text{no rebound by } t_0) &= \int_{t_0}^{t_1} \tilde{p}_{VR}(t) \\
&= \frac{\int_{t_0}^{t_1} \tilde{p}_{VR}(t)}{1 - P_{VR}(t_0)} \\
&= \frac{P_{VR}(t_1) - P_{VR}(t_0)}{1 - P_{VR}(t_0)}, \qquad \text{(D1)}
\end{aligned}
$$

for $t_1 > t_0$. Thus we can compute the desired probability directly from our cumulative probability of viral rebound, $P_{VR}(t)$.

In main text Fig. 9 we show the probability of viral rebound given $x-$day testing windows. To compute those curves, we enter t_0 as time since ATI and t_1 as the desired testing period, i.e., 1, 3, 7, 14 days in main text Fig. 9 and compute the probability of viral rebound for the next test period given a test at the time since ATI directly from eq. (D1). In Fig. S4 we show the required time-to-next-test t_1, given a test at some time post-ATI t_0, to capture a desired probability of viral rebound. To compute those curves, input t_0 and the desired probability $\text{Prob}(\text{rebound in } (t_0, t_1] | \text{no rebound by } t_0)$ into eq. (D1) and use a nonlinear solver to extract t_1.

D.2 Estimation of testing schedule from constant percent rebound curves, Figure S4

Figure D1 illustrates how we can use the next-test graph, for fixed desired probability of viral rebound, to generate an approximate testing schedule. For the purposes of this illustration we average over HIV CA-RNA levels from all study participants, since CA-RNA levels may not be measured, and assume the pre-ATI ART regimen included NNRTIs. We look for a 50% probability of viral rebound between tests. At time since ATI 0, the curve indicates that the probability of viral rebound is 50% within approximately 21 days. We then move to 21 days post-ATI, and note that given no viral rebound up to day 21, there is a 50% probability of viral rebound within approximately 7 days. We then move to 21+7=28 days post-ATI, and note that given no viral rebound up to day 28, there is a 50% probability of viral rebound within approximately 5 days. In this manner we predict a testing schedule with approximately 50% probability of viral rebound between tests, with tests at days 21, 28, 33, 37, 41, 45, and then every 3 days up to day 60.

Recall that our model is appropriate for short-term viral rebound only, which we have restricted to up to 60 days following ATI, which is the window within 210 of 235 total study participants experience viral

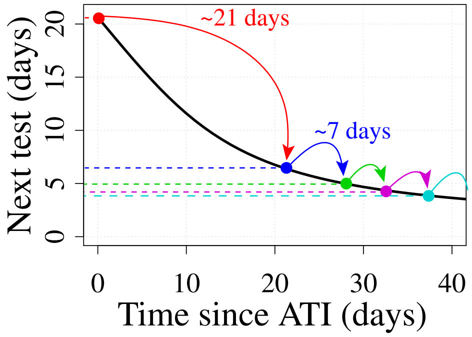

Figure D1: Visual approach to estimating a testing schedule maintaining approximately fixed inter-test rebound probability. In this figure, the time of the next test given desired 50% probability of viral rebound before the next test as a function of time since ATI, for study participants whose pre-ATI ART regimen included NNRTIs. The coloured arrows and lines are visual aides to illustrate the process of deriving the testing schedule, please see text for details.

rebound. We hypothesize that longer-term infection control, with viral rebounds up to years after ATI, is associated with mechanisms such as immune responses excluded from this model, and which remain unclear [23–25]. We therefore would recommend re-evaluation of any study participants showing no rebound by day 60.

References

[1] J.M. Conway, B.P. Konrad, and D. Coombs. Stochastic analysis of pre- and postexposure prophylaxis against HIV infection. *SIAM J Appl Math*, 73:904–928, 2013.

[2] J.M. Conway and A.S. Perelson. Residual viremia in treated HIV+ individuals. *PLoS Comput Biol*, 12:e1004677, 2016.

[3] J.M. Conway and A.S. Perelson. Early HIV infection predictions: role of viral replication errors. *SIAM J Appl Math*, 78:1863–1890, 2018.

[4] N. Chomont, M. El-Far, P. Ancuta, L. Trautmann, F.A. Procopio, B. Yassine-Diab, G. Boucher, M.-R. Boulassel, G. Ghattas, J.M. Brenchley, T.W. Schacker, B.J. Hill, D.C. Douek, J.-P. Routy, E.K. Haddad, and R.-P. Sékaly. HIV reservoir size and persistence are driven by T cell survival and homeostatic proliferation. *Nat Med*, 15:893–901, 2009.

[5] J.D. Siliciano, J. Kajdas, D. Finzi, T.C. Quinn, K. Chadwick, J.B. Margolick, C. Kovacs, S.J. Gange, and R.F. Siliciano. Long-term follow-up studies confirm the stability of the latent reservoir for HIV-1 in resting CD4+ T cells. *Nat Med*, 9:727–728, 2003.

[6] A.M. Crooks, R. Bateson, A.B. Cope, N.P. Dahl, M.K. Griggs, J.D. Kuruc, C.L. Gay, J.J. Eron, D.M. Margolis, R.J. Bosch, and N.M. Archin. Precise quantitation of the latent HIV-1 reservoir: Implications for eradication strategies. *J Infect Dis*, 212:1361–1365, 2015.

[7] Caroline Bazzoli, Vincent Jullien, Clotilde Le Tiec, Elisabeth Rey, France Mentré, and Anne-Marie Taburet. Intracellular pharmacokinetics of antiretroviral drugs in HIV-infected patients, and their correlation with drug action. *Clinical Pharmacokinetics*, 49(1):17–45, jan 2010.

[8] J.Z. Li, B. Etemad, H. Ahmed, E. Aga, R.J. Bosch, J.W. Mellors, D.R. Kuritzkes, M.M. Lederman, M. Para, and R.T. Gandhi. The size of the expressed HIV reservoir predicts timing of viral rebound after treatment interruption. *AIDS*, 30:343–353, 2016.

[9] A.S. Perelson, A.D. Neumann, M. Markowitz, J.M. Leonard, and D.D. Ho. HIV-1 dynamics in vivo: Virion clearance rate, infected cell life-span, and viral generation time. *Science*, 271:1582–1586, 1996.

[10] A.S. Perelson, P. Essunger, Y. Cao, M. Vesanen, A. Hurley, K. Saksela, M. Markowitz, and D.D. Ho. Decay characteristics of HIV-1 infected compartments during combination therapy. *Nature*, 387:188–191, 1997.

[11] J.M. Hataye, J.P. Casazza, K. Best, J. Liang, D.R. Ambrozak, S. Darko, A.R. Henry, F. Laboune, F. Maldarelli, T.T. Immonen, D.C. Douek, N.W. Hengartner, T. Yamamoto, B.F. Keele, A.S. Perelson, and R.A. Koup. Principles governing establishment versus collapse of HIV-1 cellular spread. *submitted*, 2018.

[12] R. Ke, J. Elliott, S.R. Lewin, and A.S. Perelson. Modeling the effects of vorinostat in vivo reveals both transient and delayed HIV transcriptional activation and minimal killing of latently infected cells. *PLoS Pathog*, 11:e10052371, 2015.

[13] J.E. Pearson, P. Krapivsky, and A.S. Perelson. Stochastic theory of early viral infection: Continuous versus burst production of virions. *PLoS Comput Biol*, 7:e1001058, 2011.

[14] Daniel I S Rosenbloom, Alison L Hill, S Alireza Rabi, Robert F Siliciano, and Martin A Nowak. Antiretroviral dynamics determines HIV evolution and predicts therapy outcome. *Nat Med*, 18(9):1378–1385, sep 2012.

[15] J. Shuter. Forgiveness of non-adherence to HIV-1 antiretroviral therapy. *J Antimicrob Chemother*, 61:769–773, 2008.

[16] Lin Shen, Susan Peterson, Ahmad R Sedaghat, Moira A McMahon, Marc Callender, Haili Zhang, Yan Zhou, Eleanor Pitt, Karen S Anderson, Edward P Acosta, and Robert F Siliciano. Dose-response curve slope sets class-specific limits on inhibitory potential of anti-HIV drugs. *Nat Med*, 14(7):762–766, jul 2008.

[17] FDA. *Sustiva (efavirenz): Full prescribing information*, 2016. Reference ID: 3910113.

[18] K. Murphy and C. Weaver. *Janeway's Immunobiology*. W. W. Norton & Company, 2016.

[19] V. V. Ganusov and R. J. De Boer. Do most lymphocytes in humans really reside in the gut? *Trends Immunol*, 28:514–518, 2007.

[20] C.V. Fletcher, K. Staskus, S.W. Wietgrefe, M. Rothenberger, C. Reilly, J.G. Chipman, G.J. Beilman, A. Khoruts, A. Thorkelson, T.E. Schmidt, J. Anderson, K. Perkey, M. Stevenson, A.S. Perelson, D.C. Douek, A.T. Haase, and T.W. Schacker. Persistent HIV-1 replication is associated with lower antiretroviral drug concentrations in lymphatic tissues. *Proc Natl Acad Sci USA*, 111:2307–2312, 2014.

[21] Courtney V. Fletcher and Anthony T. Podany. Antiretroviral Drug Penetration into Lymphoid Tissue. In *Encyclopedia of AIDS*, pages 1–9. Springer New York, New York, NY, 2015.

[22] H. J. Ribaudo, D. W. Haas, C. Tierney, R. B. Kim, G. R. Wilkinson, R. M. Gulick, D. B. Clifford, C. Marzolini, C. V. Fletcher, K. T. Tashima, D. R. Kuritzkes, and E. P. Acosta. Pharmacogenetics of plasma efavirenz exposure after treatment discontinuation: An adult AIDS Clinical Trials Group study. *Clin Infect Dis*, 42:401–407, 2006.

[23] B. Etemad, X. Sun, Y. Wen, G. Namazi, M.F. Kearney, W. Shao, Z.L. Brumme, D. MacMillan, R. Getz, H. Ahmed, E. Aga, R.J. Bosch, J. Jacobson, M. Carrington, R.T. Gandhi, M.M. Lederman, X.G. Yu, and J.Z. Li. HIV genetic and immune profiles of post-treatment controllers. *submitted*, 2017.

[24] A. Sáez-Cirión, C. Bacchus, L. Hocqueloux, V. Avettand-Fènoël, I. Girault, C. Lecuroux, V. Potard, P. Versmisse, A. Melard, T. Prazuck, B. Descours, J. Guergnon, J.-P. Viard, F. Boufassa, O. Lambotte, C. Goujard, La. Meyer, D. Costagliola, A. Venet, G. Pancino, B. Autran, C. Rouzioux, and the ANRS VISCONTI Study Group. Post-treatment HIV-1 controllers with a long-term virological remission after the interruption of early initiated antiretroviral therapy ANRS VISCONTI study. *PLoS Pathog*, 9:e1003211, 2013.

[25] J. M. Conway and A. S. Perelson. Post-treatment control of HIV infection. *Proc Natl Acad Sci USA*, 112:5467–5472, 2015.
