# Supplementary material for: Predictions of time to HIV viral rebound following ART suspension that incorporate personal biomarkers
Source: PLoS Comput Biol. 2019 Jul 24;15(7):e1007229. doi: 10.1371/journal.pcbi.1007229 (PMC6682162; doi:10.1371/journal.pcbi.1007229)
Supplement: S1 Table — Parameter estimates for main text model (1) with 95% confidence intervals indicated parenthetically, making no distinction between participants based on pre-ATI ART regimen. (PDF) [file pcbi.1007229.s002.pdf]

Table S1: Parameter estimates for main text model (1) with 95% confidence intervals indicated parenthetically, making no distinction between participants based on pre-ATI ART regimen.

		Detection delay type					
Shared parameters		Fixed	Exponential	Gamma	Lognormal	Weibull	Log-logistic
$as(1-q_0)$		0.04 (0.03,0.06)	0.08 (0.04,0.1534)	0.29 (0.004,19.745)	0.08 (0.03, 0.22)	0.25 (0.01, 2.78)	0.07 (0.03, 0.14)
Detection delay parameters		–	–	–	–	–	–
Fixed ($\delta-$distr.) delay	t_{delay}	4.6 (3.4,6.3)	–	–	–	–	–
Exponentially distr. delay	rate λ	–	0.10 (0.06, 0.17)	–	–	–	–
Gamma distr. delay	shape α	–	–	2.09 (0.87,5.01)	–	–	–
	rate β	–	–	0.14 (0.09,0.24)	–	–	–
Lognormally distr. delay	mean μ	–	–	–	2.1 (1.4, 2.8)	–	–
	σ	–	–	–	0.74 (0.54, 1.02)	–	–
Weibull distr. delay	scale λ	–	–	–	–	16.0 (9.6, 26.4)	–
	shape κ	–	–	–	–	1.5 (1.0, 2.2)	–
Log-logistic distr. delay	scale α	–	–	–	–	–	6.9 (2.0, 9.5)
	shape β	–	–	–	–	–	2.4 (1.5, 3.6)
**Akaike information criterion (AIC)**		180.3	176.4	174.6	178.2	175.4	179.8
