# Supplementary material for: Predictions of time to HIV viral rebound following ART suspension that incorporate personal biomarkers
Source: PLoS Comput Biol. 2019 Jul 24;15(7):e1007229. doi: 10.1371/journal.pcbi.1007229 (PMC6682162; doi:10.1371/journal.pcbi.1007229)
Supplement: S2 Table — Parameter estimates for main text model (2) with 95% confidence intervals indicated parenthetically, distinguishing study participants based on pre-ATI ART regimen. (PDF) [file pcbi.1007229.s003.pdf]

Table S2: Parameter estimates for main text model (2) with 95% confidence intervals indicated parenthetically, distinguishing study participants based on pre-ATI ART regimen.

		Detection delay type					
Shared parameters		Fixed	Exponential	Gamma	Lognormal	Weibull	Log-logistic
$as(1-q_0)$ (per day)		0.10 (0.06, 0.17)	0.14 (0.06,0.30)	0.22 (0.05,0.67)	0.18 (0.04,0.53)	0.24 (0.04,1.21)	0.16 (0.05, 0.50)
k (per day)		0.03 (0.02, 0.07)	0.03 (0.01,0.07)	0.019 (0.005,0.076)	0.02 (0.01,0.072)	0.02 (0.003,0.07)	0.02 (0.01,0.07)
Detection delay parameters		–	–	–	–	–	–
Fixed ($\delta-$distr.) delay	t_{delay}	4.2 (2.7,6.2)	–	–	–	–	–
Exponentially distr. delay	rate λ	–	0.19 (0.10,0.36)	–	–	–	–
Gamma distr. delay	shape α	–	–	2.54 (0.78,5.80)	–	–	–
	rate β	–	–	0.37 (0.12,1.11)	–	–	–
Lognormally distr. delay	mean μ	–	–	–	1.7 (1.2,2.4)	–	–
	σ	–	–	–	0.57 (0.33,0.96)	–	–
Weibull distr. delay	scale λ					7.8 (4.4,13.6)	–
	shape κ	–	–	–	–	1.7 (1.0,2.9)	–
Log-logistic distr. delay	scale α	–	–	–	–	–	5.2 (2.8,9.4)
	shape β	–	–	–	–	–	3.3 (1.7,6.3)
**Akaike information criterion (AIC)**		156.1	155.7	155.1	155.7	154.8	156.3
