# Supplementary material for: Predictions of time to HIV viral rebound following ART suspension that incorporate personal biomarkers
Source: PLoS Comput Biol. 2019 Jul 24;15(7):e1007229. doi: 10.1371/journal.pcbi.1007229 (PMC6682162; doi:10.1371/journal.pcbi.1007229)
Supplement: S3 Table — Median and 99% confidence interval in the fraction of ATI study participants showing no rebound by each test time post-ATI, computed over 10000 simulations. See text for algorithm generating the survival curves from which these were derived. For the purposes of illustration we assume 100 study participants. (PDF) [file pcbi.1007229.s004.pdf]

Table S3: Median and 99% confidence interval in the fraction of ATI study participants showing no rebound by each test time post-ATI, computed over 10000 simulations. See text for algorithm generating the survival curves from which these were derived. For the purposes of illustration we assume 100 study participants.

		Time since ATI (weeks)															
		0.5	1	1.5	2	2.5	3	3.5	4	4.5	5	5.5	6	6.5	7	7.5	8
Every two weeks, NNRTI-	.5th percentile	–	–	–	1.00	–	–	–	0.14	–	–	–	0.04	–	–	–	0.03
	Median	–	–	–	1.00	–	–	–	0.25	–	–	–	0.11	–	–	–	0.11
	99.5th percentile	–	–	–	1.00	–	–	–	0.36	–	–	–	0.19	–	–	–	0.19
Every two weeks, NNRTI+	.5th percentile	–	–	–	1.00	–	–	–	0.70	–	–	–	0.20	–	–	–	0.06
	Median	–	–	–	1.00	–	–	–	0.81	–	–	–	0.31	–	–	–	0.13
	99.5th percentile	–	–	–	1.00	–	–	–	0.90	–	–	–	0.44	–	–	–	0.23
Weekly, NNRTI-	.5th percentile	–	1.00	–	0.54	–	0.14	–	0.05	–	0.04	–	0.03	–	0.03	–	0.03
	Median	–	1.00	–	0.66	–	0.25	–	0.12	–	0.11	–	0.11	–	0.11	–	0.11
	99.5th percentile	–	1.00	–	0.78	–	0.36	–	0.22	–	0.19	–	0.19	–	0.19	–	0.19
Weekly, NNRTI+	.5th percentile	–	1.00	–	0.93	–	0.70	–	0.41	–	0.20	–	0.10	–	0.06	–	0.04
	Median	–	1.00	–	0.98	–	0.81	–	0.54	–	0.31	–	0.19	–	0.13	–	0.11
	99.5th percentile	–	1.00	–	1.00	–	0.90	–	0.66	–	0.44	–	0.30	–	0.23	–	0.21
Twice weekly, NNRTI-	.5th percentile	1.00	0.83	0.54	0.29	0.14	0.07	0.05	0.04	0.04	0.03	0.03	0.03	0.03	0.03	0.03	0.03
	Median	1.00	0.91	0.66	0.41	0.25	0.16	0.12	0.11	0.11	0.11	0.11	0.11	0.11	0.11	0.11	0.11
	99.5th percentile	1.00	0.97	0.78	0.54	0.36	0.26	0.22	0.20	0.19	0.19	0.19	0.19	0.19	0.19	0.19	0.19
Twice weekly, NNRTI+	.5th percentile	1.00	0.98	0.93	0.83	0.70	0.55	0.41	0.29	0.20	0.14	0.10	0.07	0.06	0.04	0.04	0.04
	Median	1.00	1.00	0.98	0.91	0.81	0.67	0.54	0.41	0.31	0.24	0.19	0.16	0.13	0.12	0.11	0.11
	99.5th percentile	1.00	1.00	1.00	0.98	0.90	0.79	0.66	0.54	0.44	0.35	0.30	0.26	0.23	0.21	0.21	0.20
