# Supplementary figures and images for: Predictions of time to HIV viral rebound following ART suspension that incorporate personal biomarkers
Source: PLoS Comput Biol. 2019 Jul 24;15(7):e1007229. doi: 10.1371/journal.pcbi.1007229 (PMC6682162; doi:10.1371/journal.pcbi.1007229)

Prob. Density

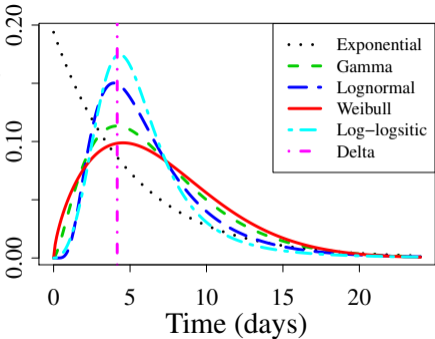

Supplement: S1 Fig — Model-predicted probability density functions for delay between activation that induces rebound and detectable viremia, D(t), parameters estimated for main text Eq (2). Probability density formulas are provided in main text Table 1 and parameter estimates for the probability densities are given in Table. (PDF) [file pcbi.1007229.s005.pdf]

(a)

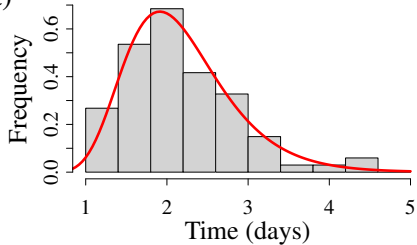

(b)

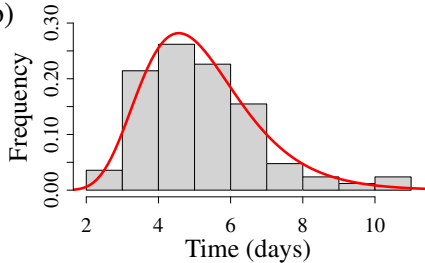

Supplement: S2 Fig — Histograms of model-predicted average time between successful latent cell activations, across ATI study population (see main text, Description of data), assuming (a) a Weibull-distributed detection delay and (b) a fixed detection delay, with lognormal distribution fit shown in red. The lognormal distribution fits is best when compared to some stylized fits (tested exponential, Weibull, and Burr distributions, ΔAIC > 4.5) but statistically indistinguishable from others (tested gamma and log-logistic distributions, 2 < ΔAIC < 3). The population-average is predicted to be a successful activation every (a) 2.2 days, (b) 5.2 days. (PDF) [file pcbi.1007229.s006.pdf]

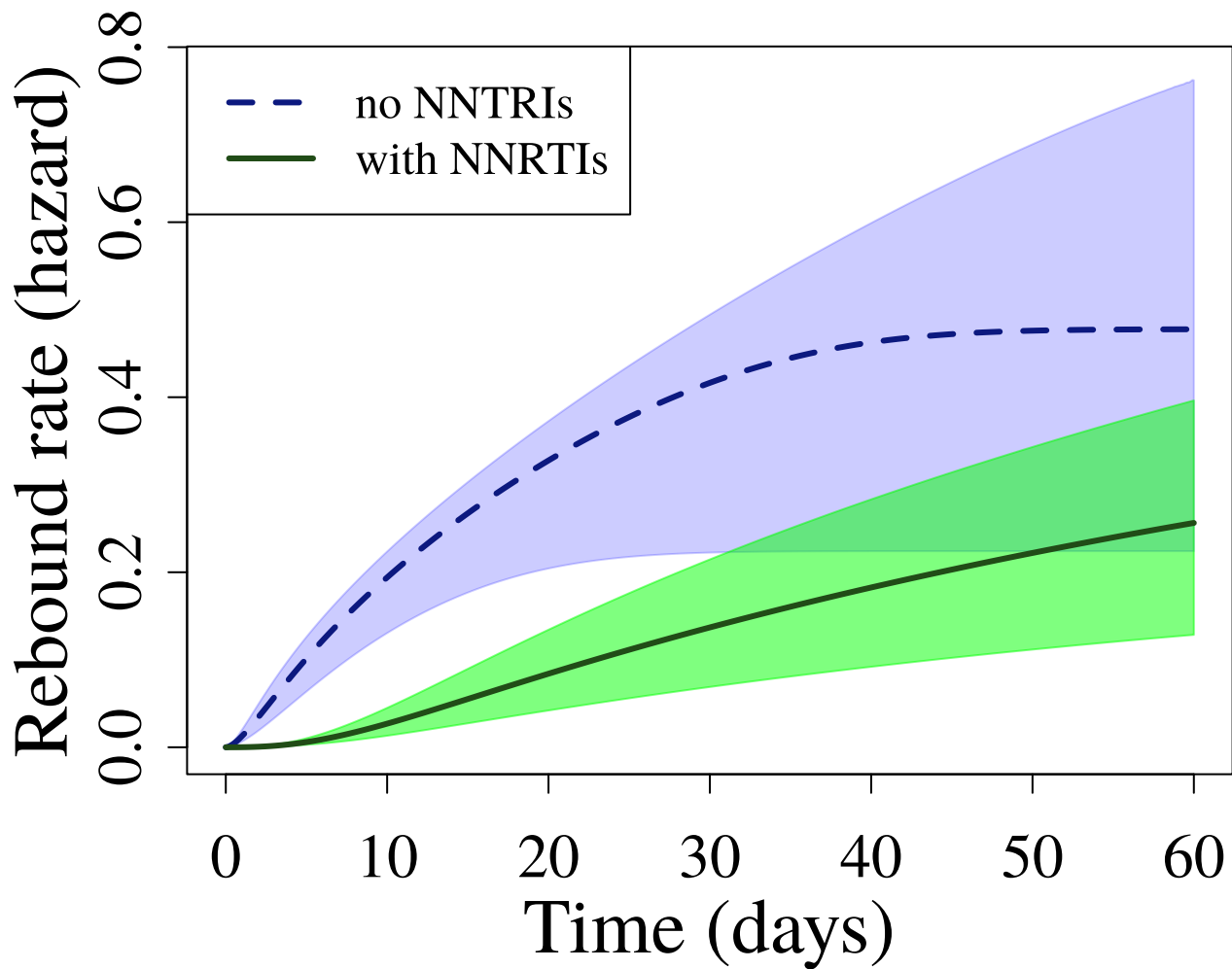

Supplement: S3 Fig — Areas indicate span of predicted rebound rates depending on pre-ATI HIV CA-RNA level across all data, with green indicating data from study participants who included NNRTIs in their pre-ATI ART regimen, and blue indicating study participants who did not. The solid/dashed lines indicate the rebound rates assuming median HIV CA-RNA levels for each group. (PDF) [file pcbi.1007229.s007.pdf]

(a)

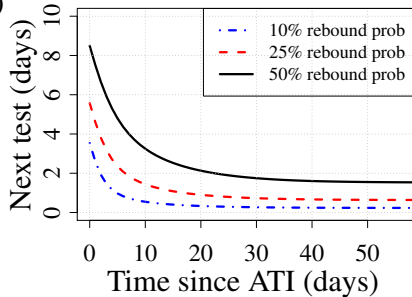

(b)

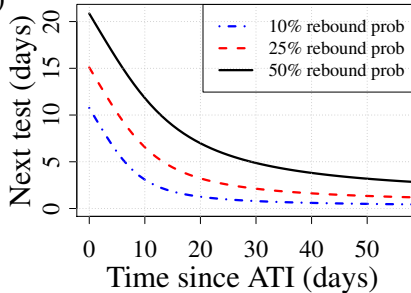

Supplement: S4 Fig — Model-recommended frequency of testing for ATI clinical trials, depending on study objectives, averaged over study participant HIV CA-RNA levels. Next-test time given a fixed, desired, probability of viral rebound, as a function of time since ATI, for study participants whose pre-ATI ART regimen (a) excluded and (b) included NNRTIs. (PDF) [file pcbi.1007229.s008.pdf]

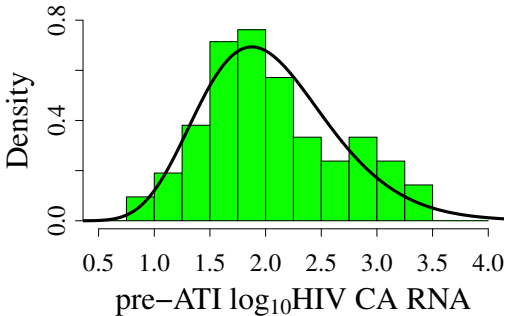

Supplement: S5 Fig — Histograms of log10(HIV CA RNA) levels from [2] (see main text, Description of data), with gamma distribution fit. The gamma distribution fits is best when compared to some stylized fits (tested Weibull, log-normal, log-logistic, and Burr distribution) yielding the lowest log-likelihood. Since all tested distributions have the same number of parameters, we have no need to call on the AIC. (PDF) [file pcbi.1007229.s009.pdf]

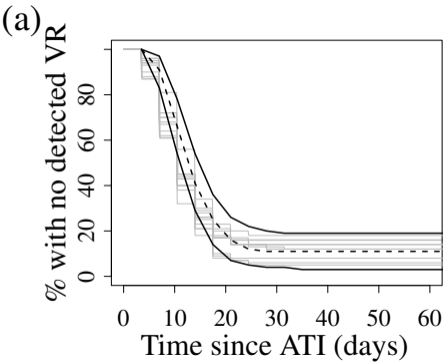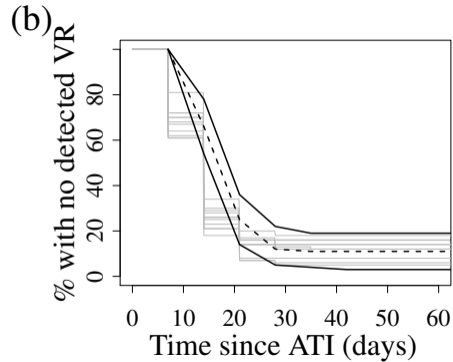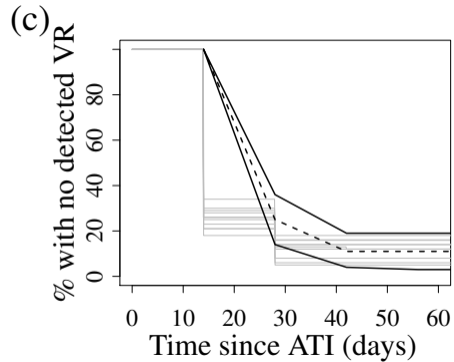

Supplement: S6 Fig — Ten sample survival curves for in silico studies with 100 study participants whose pre-ATI ART regimen excluded NNRTIs. Median (dashed line) and and 99% confidence interval (solid lines) computed from 10000 simulations. Survival curves describe model-predicted time to detectable viral rebound, given a post-ATI viral load testing schedule with (a) twice-weekly, (b) weekly, or (c) every two week testing. (PDF) [file pcbi.1007229.s010.pdf]
